# Mechanisms leading to occupational oral exposure: a systematic review and meta-analysis

**DOI:** 10.1093/annweh/wxaf042

**Published:** 2025-07-25

**Authors:** Marlene Dietz, Anke Kahl, Urs Schlüter

**Affiliations:** Unit 4.I.4 Exposure Assessment, Exposure Science, Division 4 Hazardous Substances and Biological Agents, Federal Institute for Occupational Safety and Health (BAuA), Dortmund, Germany; Chair of Occupational Safety, School of Mechanical Engineering and Safety Engineering, University of Wuppertal, Wuppertal, Germany; Chair of Occupational Safety, School of Mechanical Engineering and Safety Engineering, University of Wuppertal, Wuppertal, Germany; Unit 4.I.4 Exposure Assessment, Exposure Science, Division 4 Hazardous Substances and Biological Agents, Federal Institute for Occupational Safety and Health (BAuA), Dortmund, Germany

**Keywords:** aerosols, inadvertent ingestion, mechanisms, occupation, oral exposure, PRISMA, transfer parameters, workplace

## Abstract

**Introduction:**

In addition to inhalation and dermal exposure, also oral exposure is relevant in the workplace, even though this exposure route is most often neglected. In order to improve the understanding of occupational oral exposure, a systematic identification of mechanisms leading to inadvertent ingestion in the workplace is needed, including the transfer of chemicals and the contribution of aerosols.

**Methods:**

A systematic literature search was conducted according to the PRISMA method including 5 databases and 9 institutional websites. Information from the included studies was extracted in concept matrices and further analyzed.

**Results:**

Overall, 175 suitable publications were selected. Identified mechanisms leading to oral exposure were the transfer of chemicals, eg, from hands to mouth, and contributions from aerosols. Transfer influencing parameters were categorized as environmental, substance-specific, transfer pathway, surface, contact, or skin characteristics.

**Discussion:**

Even though oral exposure was mainly investigated for children so far, similar mechanisms can lead to oral exposure in adults. Although the parameters characterizing the transfer of chemicals were identified and categorized, inconsistencies in nomenclature were identified, and correlations between parameters and transfer efficiencies often remained unclear.

**Conclusions:**

The transfer of hazardous substances and the contribution of aerosols were identified as mechanisms of oral exposure. Transfer parameters and the interplay between particles from aerosols and oral exposure were discussed.

**What’s Important About This Paper?**

Recent research has shown that oral exposure is relevant in the workplace, though it is rarely considered in current risk assessments. This systematic literature search and meta-analysis provides a comprehensive summary of the mechanisms leading to oral exposure and thus the basis for further development of occupational oral exposure assessment.

## Introduction

Most often, inhalation and dermal exposure are assessed to protect workers dealing with hazardous substances. Recent research demonstrated that oral exposure can be of relevance in different workplaces (Dietz et al. 2023). Therefore, oral exposure in the workplace also needs consideration for an overall protection of workers.

To ensure meaningful assessments of this exposure route, it is necessary to understand the mechanisms leading to unintended ingestion in the workplace. The knowledge and understanding of these mechanisms are also a prerequisite for purposeful and simple modeling approaches as well as for implementation effective measures for worker protection.

So far, many approaches do not focus on workplaces but on consumer protection and especially on small children. Hand-to-mouth contacts are often assessed as the main mechanism (Li et al. 2023; Zhang et al. 2023) or a constant ingestion rate specific for age but not for situations or tasks is assumed (Li et al. 2019, 2021; Dietz et al. 2023). In contrast to this, Gorman Ng et al. developed a workplace model, which describes the transfer pathway between surfaces and mouth as the main mechanism based on contact frequencies (Gorman Ng et al. 2012, 2016).

A comprehensive and systematic identification of mechanisms leading to the emergence of oral exposure is missing so far. Especially parameters influencing the transfer of chemicals and the contribution of aerosols to oral exposure need further investigation.

Thus, the aim of this study is to systematically identify mechanisms, which lead to occupational oral exposure. Therefore, (i) mechanisms leading to oral exposure were systematically collected, and their relevance was evaluated in the context of work. (ii) Parameters that characterize the transfer of chemicals potentially leading to oral exposure were categorized, and research gaps regarding transfer efficiencies were identified. (iii) The current state of knowledge on aerosols contributing to oral exposure was processed for future modeling approaches.

## Methods

To systematically address these 3 objectives, a systematic literature review was conducted according to the Preferred Reporting Items for Systematic reviews and Meta-Analyses (PRISMA) method (Page et al. 2021). In preparation for the following meta-analysis, data were extracted using concept matrices. There was no review protocol or registration of this study.

### Information sources and search strategies

As data sources for the review, the 5 databases Web of Science, PubMed, COCHRANE, bergischbib, and Deutsche Nationalbibliothek were selected. Additionally, websites from 9 research institutes were searched to identify further relevant reports not included in the databases. An overview of the data sources is given in Supplementary Table S1.

To address the 3 objectives, 3 distinctive search strategies were defined. (i) Looking for information on any mechanisms of oral exposure, synonyms of “oral,” “exposure,” and “mechanism” were combined. Specific word families were excluded to specify the focus, eg, to exclude medicinal treatments or diet-specific information. (ii) To address information on the transfer of chemicals, synonyms of “transfer efficiency” and different zones as hands or arms were included. Biological substances and heat transfer were excluded because they were not in focus of this study. (iii) To identify studies on the interplay of oral exposure and aerosols, synonyms for “oral exposure” and “aerosol” were combined.

To evaluate the 3 main search strategies, 5, 6, and 2 evaluation publications were selected, respectively (Supplementary Table S2). These publications are examples of suitable studies for assessing the research question and were known to the authors prior to the systematic literature search. It was then tested whether they were identified by the 3 strategies in the databases Web of Science and PubMed, as most of the evaluation publications are available here (Supplementary Table S2). For the first search strategy, 4 and 4 evaluation publications were generally available in Web of Science and PubMed, respectively. Of these, 4 publications were identified in each database. For the second search strategy, all evaluation publications were available in both databases and were identified by the search strategy. For the third strategy, all evaluation publications were available and identified in both databases. The 3 final search strategies are summarized in Supplementary Table S3. In addition to these strategies, especially websites need simpler search requests and therefore, simplified or in specific cases also analogous German search strategies were defined and documented in Supplementary Table S4, including also the latest date of search.

### Study selection

The study selection is based on the methodology described in former publications (Dietz et al. 2023, 2025) and includes the definition of suitability criteria for title/abstract and full text level as well as the evaluation of the corresponding criteria in consistency checks.

On the title and abstract level, for search strategy (i), the Population was defined as children, adults, or workers, as general mechanisms of oral exposure were of interest. The Outcome criteria were any investigations on the emergence of oral exposure. Thereby, eating, drinking, and medical uptake were excluded as these are intentional processes. Publications describing oral exposure to a substance without naming the corresponding mechanism of oral exposure were also excluded. On the full text level, the Outcome criteria were narrowed so that concrete mechanisms leading to oral exposure needed to be described.

For search strategy (ii), on the title and abstract level, the Population was defined as any chemical substance. The Outcome needed to be any information on transfer investigations. On the full text level, the Outcome criterion was narrowed so that either parameters influencing the transfer or quantitative information on transfer efficiencies needed to be included.

On the title and abstract level of search strategy (iii), the Population needed to be adults or workers, as it was assumed that body size may matter for the interplay of aerosols and the emergence of oral exposure. Furthermore, information on chemical and biological substances in aerosols was accepted to not exclude relevant information. However, the transferability between biological and chemical substances is not warranted. The Outcome criteria were defined as any information on the interplay between aerosols and oral exposure. On the full text level, the Outcome criteria were specified as either qualitative or quantitative information.

### Data extraction and data analysis

For the extraction of qualitative and quantitative data, a concept matrix was used (Webster and Watson 2002). The methodology is similar to former systematic reviews (Dietz et al. 2023, 2025).

The concept matrix includes general information on the substance group, context, and persons as well as detailed categories on the 3 objectives: (i) mechanisms, (ii) transfer, and (iii) aerosols.

## Results

### Study selection and included studies

Preparing the study selection, the title and abstract, and full text criteria were evaluated in parallel blind ratings of 2 reviewers (consistency checks). For the first screening level, Cohen’s kappa was calculated as 0.429, which can be interpreted as moderate agreement according to Landis and Koch (Landis and Koch 1977). Therefore, the criteria were critically discussed by both reviewers and specified, leading to the criteria given above. The reviewers resolved their disagreements by consensus and agreed that the criteria specification was sufficient for a consistent application of criteria. On the full text level, Cohen’s kappa was 0.048, which can be interpreted as slight agreement according to Landis and Koch (Landis and Koch 1977). This low level of agreement mainly resulted from 1 major misunderstanding between both reviewers, how to rate the Population criteria if none of the 3 Outcome criteria were fulfilled. As this was identified as a major cause of different ratings by the reviewers, a clarification was deemed to be sufficient without repetition of the consistency check.

By applying this methodology, 15,774 studies were initially identified. Of these, 13,251 remained after duplicate removal and were automatically sorted by their relevance based on the occurrence of predefined keywords:

Population: “child,” “adult,” “worker,” “chemical,” “biological”;
Outcome: “mechanism,” “ingestion,” “oral,” “transfer,” “efficienc,” “aerosol,” “gastrointestinal,” “clearance”.

**Fig. 1.**
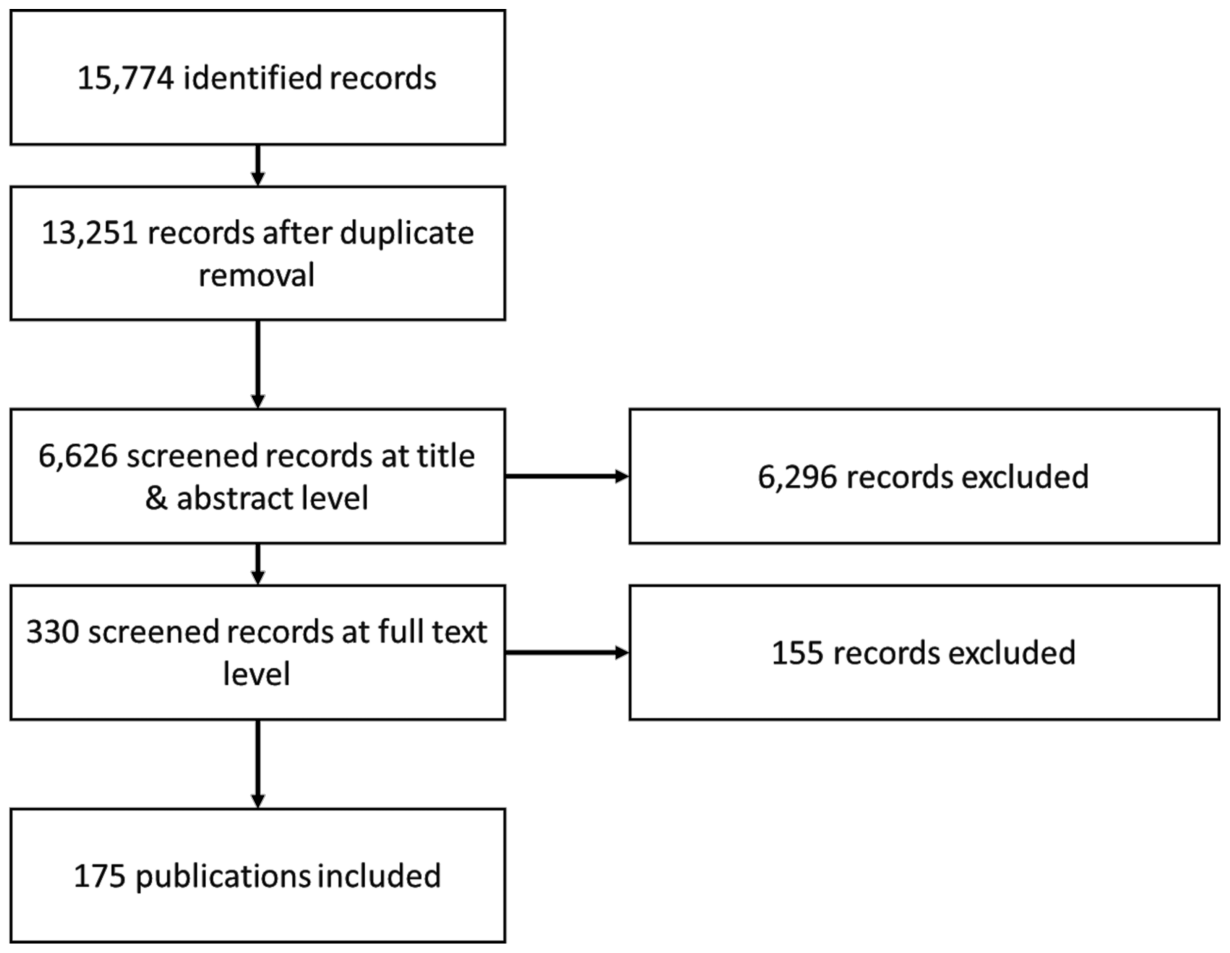
Flowchart for the identification and screening of the publications.

Based on this, the most relevant 6,626 publications were screened on title and abstract level, and 330 publications were screened on the full text level. Finally, 175 publications were identified as suitable, and the corresponding data were extracted. A summary is given in the flow chart in Fig. 1. An overview is given in Supplementary Table S5 of the included studies with information on the extracted data categories.

The different Population and Outcome categories are summarized to get a better overview of the available database. Most publications describe mechanisms of oral exposure in general (*n* = 140), followed by publications describing transfer parameters (*n* = 62) or aerosols (*n* = 25) contributing to oral exposure. Furthermore, most of the publications investigated oral exposure of children (*n* = 88). Less studies were available for adults (*n* = 68), and even less for workers (*n* = 42).

Outdoor spaces, indoor rooms, and simulated scenarios were described in the publications. Furthermore, biological substances, metals, and pesticides were addressed most frequently. More detailed information on the substance groups and contexts is given in Supplementary Figs. S1 and S2.

### Mechanisms of oral exposure

Especially age contributes to behavior and thereby to different mechanisms of oral exposure. Therefore, studies addressing mechanisms of oral exposure distinguish between children (*n* = 87) and adults (*n* = 56). The most frequent mechanism identified is contacting the mouth with the hand (*n* = 122). Further mechanisms are object-mouth contacts (adult-specific wording, *n* = 16) and mouthing (child-specific wording, *n* = 26). Additionally, the fecal-oral path (*n* = 8), deposition on or contamination of the perioral area (*n* = 10), and contributions of aerosols (*n* = 19) were identified. Furthermore, the publications identified personal behavior as smoking (*n* = 3) and nail chewing (*n* = 8) as relevant mechanisms. Thus, this first part of the results also confirms that the transfer of chemicals and aerosols can contribute to oral exposure.

### Transfer of chemical substances

The studies on the transfer of chemical substances described different parameters for its characterization. These parameters can be grouped in 6 coarse categories as influencing factors:

substance properties (*n* = 37),
surface characteristics (*n* = 29),
characteristics of the contact (*n* = 36),
environmental parameters (*n* = 10),
transfer path (start and destination, *n* = 6) and
skin properties (*n* = 22).

**Fig. 2.**
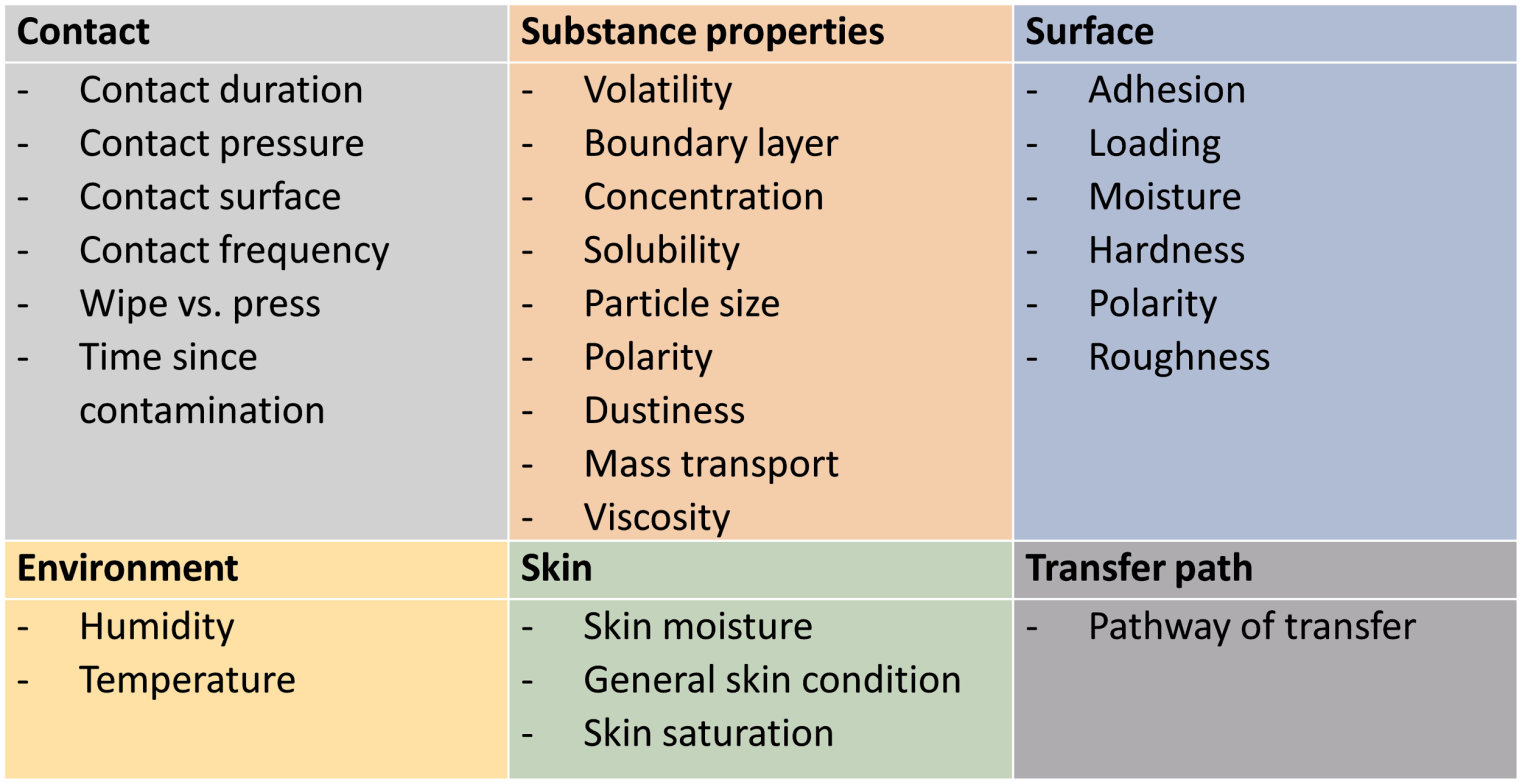
Six categories of transfer parameters with the included parameters as they are described in the individual studies.

These coarse categories include more detailed information, as illustrated in Fig. 2.

For each of these parameters, it was extracted whether an increase of the parameter results in an increase (positive correlation), no change (no correlation), decrease (negative correlation), or unclear change (unclear correlation) of the transfer efficiency. Each of these coarse categories, with its corresponding details, was investigated using this additional information.

**Fig. 3.**
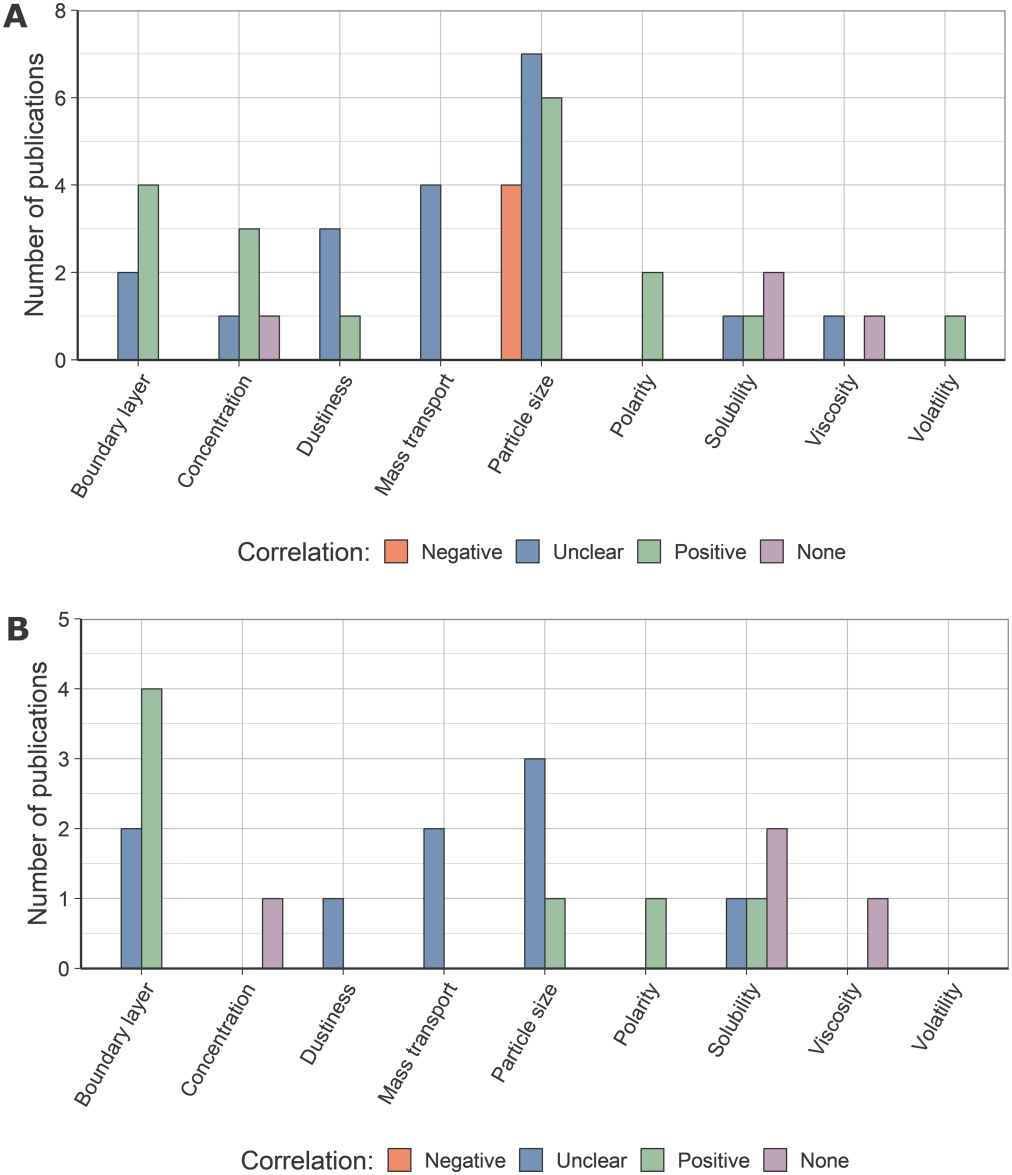
(A) Substance-related transfer parameters for whole database. (B) Substance-related transfer parameters for database, which explicitly refer to transfer efficiencies. For both plots, the correlation between parameter and transfer (efficiency) is indicated according to the legend.

Exemplarily, Fig. 3A illustrates the different substance properties and their correlation with the transfer efficiency according to the included studies.

Thereby, boundary layer describes the ability of the substance to form boundary layers, which affect the transfer. The concentration, polarity, and solubility refer to the considered substance (in solution), if relevant.

For all substance properties, there were studies identified that describe either a positive or unclear correlation to the transfer efficiency. Additionally, there are single statements in publications where no correlation was found for concentration, solubility, and viscosity. There were also 4 publications, which stated a negative correlation to the transfer efficiency for the particle size. Here, varying dependencies between the surface type, especially the surface roughness, and the particle size were described (Ferguson et al. 2009; Stefaniak et al. 2021).

In addition to the different parameters, inconsistencies in the used wording among the studies were observed regarding the absolute term “transfer” and the term “transfer efficiency,” which relates the transferred mass to the overall available mass of a substance. To investigate differences between the overall database (Fig. 3A) and those publications, which directly refer to relative term “transfer efficiency,” those publications were additionally evaluated separately, as shown in Fig. 3B. After building of this subset, especially unclear and positive correlations remain for the boundary layer, dustiness, mass transport, particle size, polarity, and viscosity. For volatility, no data sets remain. Viscosity and solubility additionally contain publications with no identified correlation.

Due to the thereby excluded studies, the number of correlations per substance characteristic decreases. Especially, the positive correlations for volatility, concentration, and dustiness were completely eliminated. The unclear correlations for concentration and viscosity as well as the negative correlation for particle sizes cannot be observed in this data subset.

**Fig. 4.**
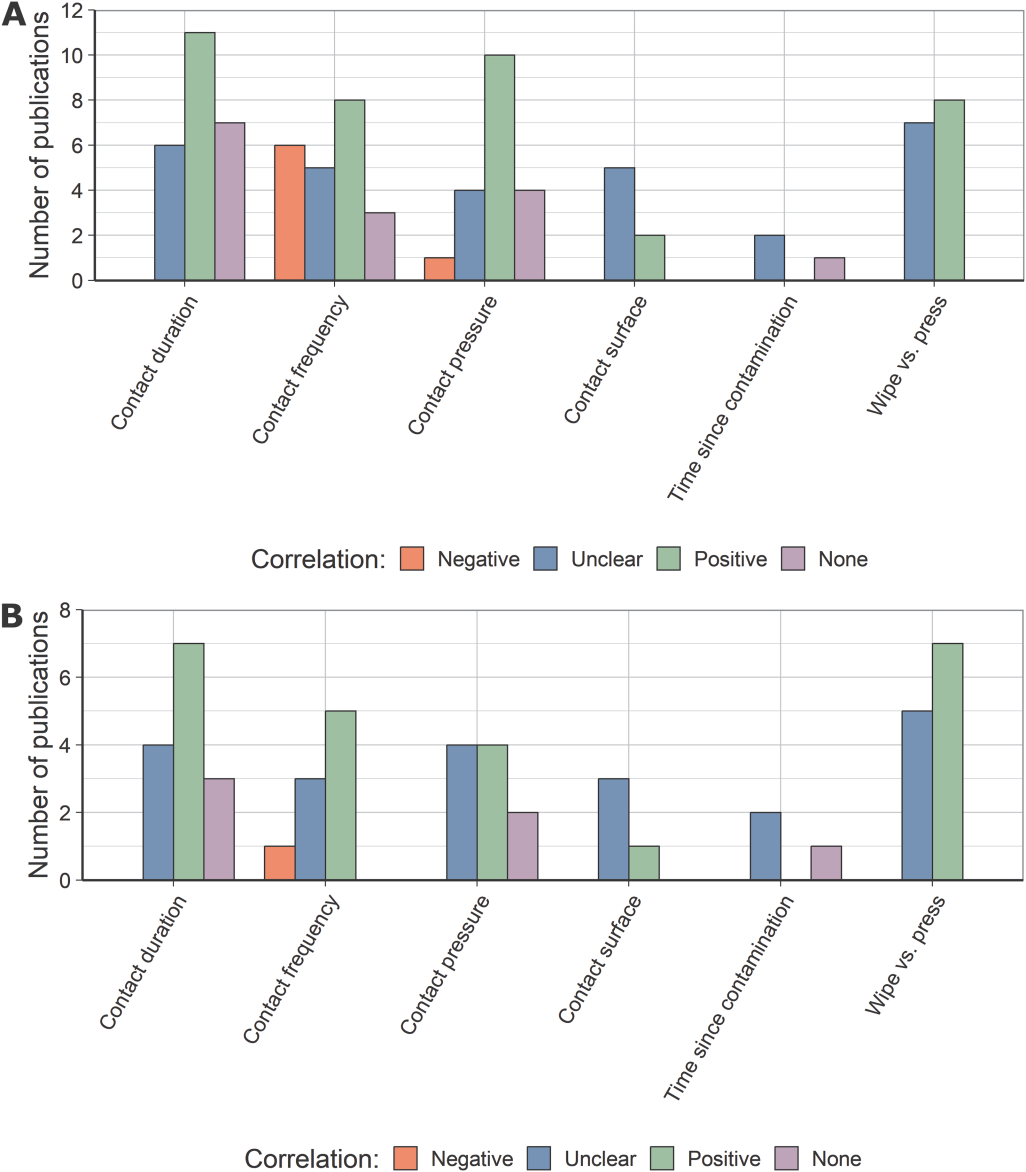
(A) Contact-related transfer parameters for the whole database. (B) Contact-related transfer parameters for the database, which explicitly refer to transfer efficiencies. For both plots, the correlation between parameter and transfer (efficiency) is indicated according to the legend.

Similar to the substance properties, contact characteristics were also analyzed. Thereby, unclear, positive, and no correlations dominate most of the categories as shown in Fig. 4A.

Negative correlations were observed for contact pressure and frequency. An equilibrium of transfer to and away from the respective surface is established, especially for the contact frequency with an increasing number of contacts (Rodes et al. 2001; Hubal et al. 2008; Goede et al. 2019). The overall correlations change for the subset of publications on “transfer efficiencies,” which were summarized in Fig. 4B. Due to the reduced dataset, the overall numbers of observed correlations were reduced. However, especially the negative correlations of contact frequency and transfer efficiency were smaller than for the overall database.

In addition to the parameters on the transfer of chemicals, 45 publications reporting values for transfer efficiencies were extracted. A list of these publications is available from Supplementary Table S5. As the focus of this study is the analysis of mechanisms of oral exposure, a further analysis of quantitative transfer efficiencies is excluded from this publication.

### Contribution from aerosols

**Fig. 5.**
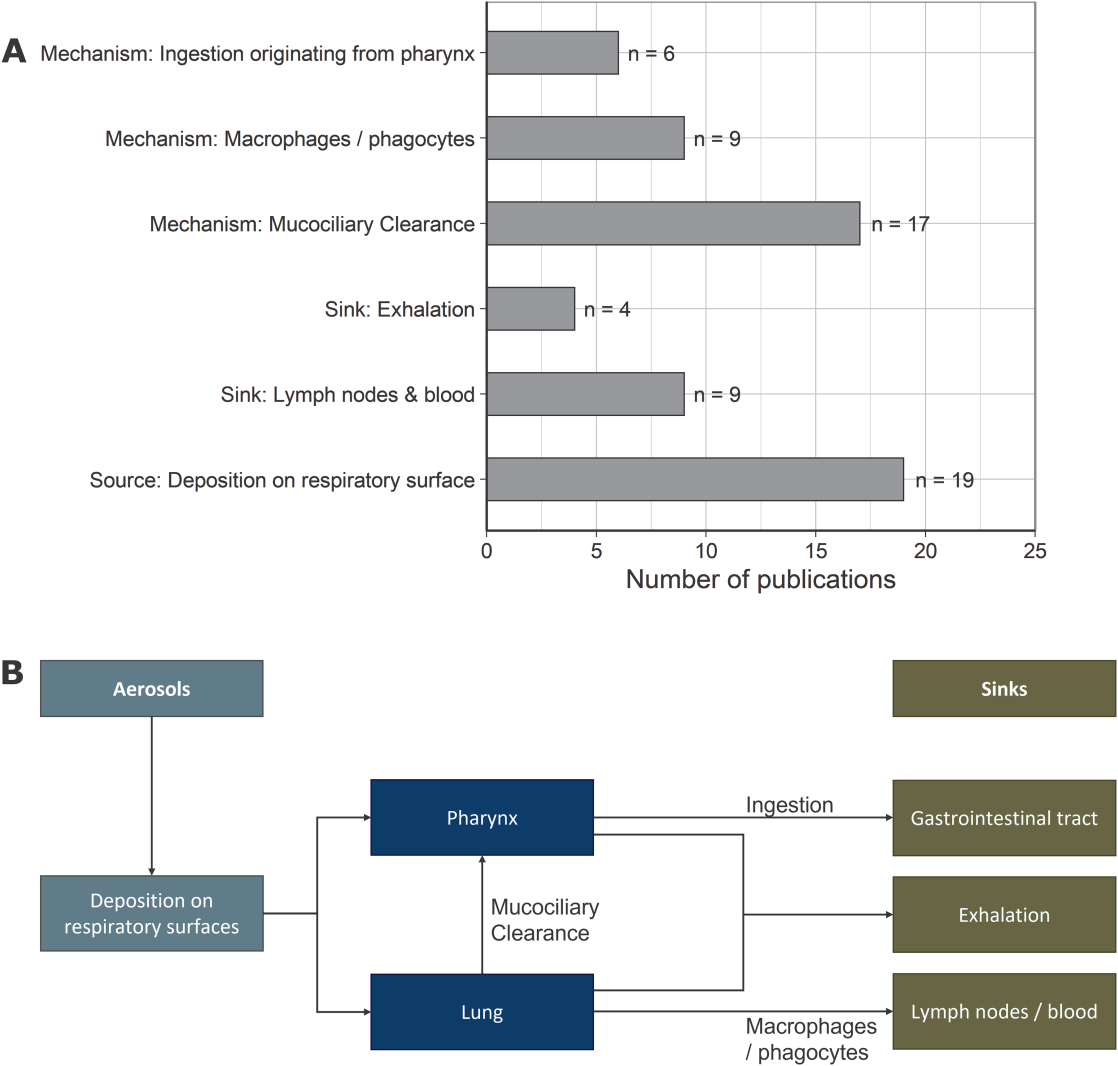
(A) Overview of relevant sources and sinks as well as mechanisms for the transfer of aerosols in the human body according to the included studies. (B) Summarized pathway of aerosol particles in the human body.

The contribution of aerosols to oral exposure was investigated in more detail. Thereby, 15 of the publications described the particle size as an important characteristic for the distribution of aerosols in the human body. Six categories were identified based on the included studies and are summarized in Fig. 5A, where the pathway of particles is illustrated in Fig. 5B.

In general, these studies describe the deposition of particles in the pharynx (oral cavity and throat) region as starting point for the swallowing of particles. Particles in the pharynx region were either deposited there directly during breathing due to their size (extrathoracic fraction). Alternatively, these particles originate from the lung, from where they were transported via mucociliary clearance (self-cleaning of the respiratory tract). Other described sinks are exhalation of particles or transport to the lymph nodes and the blood via macrophages/phagocytes.

In addition, the particle size is an important characteristic for the interplay of aerosols and oral exposure. The publications describe particles from the extrathoracic fraction as aerodynamic diameters > 10 µm. Aerodynamic diameters of particles cleared from the lung and subsequently swallowed are described as 2.5 to 10 µm.

## Discussion

Mechanisms leading to oral exposure were investigated to better understand the emergence of this exposure pathway in the workplace. Thereby, a high number of identified studies represents the recent research focus on children’s oral exposure, especially with the description of the contribution of mouthing. The frequencies of specific behaviors vary widely between children and adults. Where mouthing is a typical and frequent behavior among young children, object-mouth and hand-mouth contacts decline over age. Nevertheless, the behaviors themselves remain similar. However, as documented by Dietz et al. (Dietz *et al.* 2025), adults also contact with their face or even their mouth with their hands, although the frequencies and thereby the impact are generally lower than for children. Therefore, mechanisms based on specific behaviors can be transferred between children and adults, as long as the quantitative assessment is age-specific.

The evaluated studies describe mechanisms, which represent different ways of “transfer” including

hand-mouth contact,
object-mouth contacts (adults) or mouthing (children),
fecal-oral transmission, and
transfer between the perioral region and the mouth.
Smoking or nail chewing are specific behaviors contributing to transfer.

Furthermore, aerosols are identified as a contribution to oral exposure.

In addition to this general identification of mechanisms leading to oral exposure, parameters influencing the transfer of chemicals are identified, including their correlation with the transfer efficiency. The literature describes a variety of parameters, which are summarized into 6 categories in this work (substance properties, surface characteristics, contact characteristics, environmental parameters, transfer path, and skin properties). Surface, substance, or contact parameters are investigated most often. However, many correlations are still unclear according to the individual studies, and interactions of different parameters are barely investigated. Therefore, there is still a lack of understanding regarding conditions enhancing or reducing transfer efficiencies.

The nomenclature is inconsistent in the literature as the absolute term “transfer” and the relative term “transfer efficiency” are inconsistently used, which should be improved in future studies. Whether these inconsistencies would influence the identified correlations can hardly be determined due to the limited database.

The third aspect of this review is the influence of aerosols on oral exposure in adults. As the gastrointestinal tract is 1 potential sink for particles from aerosols entering the human body, depending on their size, aerosols should not generally be neglected in the context of oral exposure. However, the relevance compared to the transfer of substances needs further investigation as well as the interplay with the assessment of inhalation exposure. Furthermore, the contribution of precipitation of aerosols in saliva should be investigated, as this is not described in the identified publications, and saliva is frequently swallowed.

### Analysis of bias

The included studies identified mechanisms, transfer parameters, or contributions of aerosols, as the study authors assume them to be relevant for oral exposure. However, contradicting assumptions, eg, mechanisms deemed irrelevant or parameters not influencing the transfer, are most frequently not mentioned and thereby, this perspective is underrepresented in this review by design.

Furthermore, bias can occur from the results themselves. As the number of included studies is small, especially for the transfer efficiency correlations, experimental conditions from single studies can predominate the results. Therefore, apparently dominating positive correlations underly bias through the small number of studies and experimental conditions. This is, for example, illustrated by the contradicting results for the particle size, caused by interplays with study-specific surface characteristics. Thus, the results might not be representative, and misinterpretations can generally lead to bias.

### Limitations and strengths

Although the search strings and underlying databases warrant an extensive search, this study is limited to studies published between the year 2000 and now and to English or German language publications. Furthermore, only 1 researcher (M.D.) assessed the publications during title and abstract, and full text screening. However, on both screening stages, consistency checks were implemented, and the results were critically discussed to warrant self-consistent ratings during the screening process. Similarly, the data extraction was performed by only 1 researcher (M.D.). The extraction was formalized to minimize errors by concept matrices, which also emphasize a structured and gradual data extraction.

A strength of this systematic literature search is the extensive search including adults and children, to include experiences and research results for children and to abstract the results on adults (and workers), where the research base is very limited. Furthermore, the general mechanisms were systematically identified and both identified mechanisms, transfer and contributions from aerosols, were themselves systematically searched and evaluated. Additional searches on institutional websites completed the information from databases. The resulting high number of publications could be handled by sorting the studies according to keywords, which warrants the screening and inclusion of the most relevant studies.

## Conclusion

The objective of this review was to identify mechanisms leading to oral exposure, to summarize the current knowledge on transfer influencing parameters, and to systematically describe the interplay of aerosols and oral exposure. Therefore, 140, 62, and 25 studies were included on general mechanisms, transfer, and aerosols, respectively. Based on this, the transfer of chemicals (eg hand-mouth, object-mouth) as well as the contribution of aerosols are confirmed as the mechanisms of oral exposure. For the transfer of chemicals, 6 categories were developed to summarize the variety of parameters described in different studies. Regarding aerosols, the gastrointestinal tract is identified as a sink (next to the blood/lymph nodes), illustrating the interplay of particles from aerosols and oral exposure.

Finally, this review identified mechanisms leading to (occupational) oral exposure and processed the knowledge base in this regard as it is needed for further investigations of both transfer parameters and their correlations with transfer efficiencies and the contribution from aerosols.

## Supplementary material

Supplementary material is available at *Annals of Work Exposures and Health* online.

wxaf042_suppl_Supplementary_Tables_1-5_Figures_1-2

## Data Availability

The original contributions presented in the study are included in the article/supplementary material; further inquiries can be directed to the corresponding author.
