# Supplementary material for: Mechanisms leading to occupational oral exposure: a systematic review and meta-analysis
Source: Ann Work Expo Health. 2025 Jul 25;69(8):798–807. doi: 10.1093/annweh/wxaf042 (PMC12463556; doi:10.1093/annweh/wxaf042)
Supplement: wxaf042_suppl_Supplementary_Tables_1-5_Figures_1-2 [file wxaf042_suppl_supplementary_tables_1-5_figures_1-2.pdf]

Supplementary Material

Mechanisms leading to occupational oral exposure: A systematic review and meta-analysis

Marlene Dietz[1,2*], Anke Kahl[2] and Urs Schlüter[1]

1 Unit 4.I.4 Exposure Assessment, Exposure Science, Division 4 Hazardous Substances and Biological Agents, Federal Institute for Occupational Safety and Health (BAuA), Dortmund, Germany, 2 Chair of Occupational Safety, School of Mechanical Engineering and Safety Engineering, University of Wuppertal, Wuppertal, Germany,

Supplementary Table 1: Overview of included databases and institutional websites with corresponding URL.

Information source	Database	Website	URL
bergischbib	x		http://www.bergischbib.de/
COCHRANE	x		https://www.cochranelibrary.com/advanced-search
Deutsche Nationalbibliothek	x		https://katalog.dnb.de
PubMed	x		https://pubmed.ncbi.nlm.nih.gov/advanced/
Web of Science	x		https://www.webofscience.com
Federal Institute for Occupational Safety and Health (BAuA)		x	https://www.baua.de/DE/Angebote/Publikationen/Publikationen_node.html
United States Environmental Protection Agency (EPA)		x	https://www.epa.gov/nscep (Advanced search)
Health and Safety Executive (HSE)		x	https://www.hse.gov.uk/pubns/
Institute of Occupational Medicine (IOM)		x	https://www.iom-world.org/research/online-library/
National Institute for Occupational Safety and Health (NIOSH)		x	https://www2a.cdc.gov/nioshtic-2/advsearch2.asp
Organisation for Economic Co-operation and Development (OECD)		x	https://www.oecd-ilibrary.org/
National Institute for Public Health and the Environment (RIVM)		x	https://www.rivm.nl/en/recentpublications
Netherlands Organisation for Applied Scientific Research (TNO)		x	https://repository.tno.nl/islandora/search/
World Health Organization (WHO)		x	https://apps.who.int/iris/

Supplementary Table 2: Overview of publications used for the evaluation of search strategies and evaluation results for Web of Science and PubMed.

No	Search strategy	Source	Web of Science		PubMed	
			Available	Included	Available	Included
1	1	(Gorman Ng et al. 2012)	x	x	x	x
2	1	(Gorman Ng et al. 2016)	x	x	x	x
3	1	(Deubner et al. 2001)	-	-	x	-
4	1	(Cherrie et al. 2006)	x	x	x	x
5	1	(Hubbard et al. 2022)	x	x	x	x
6	2	(Gorman Ng et al. 2014)	x	x	x	x
7	2	(Sahmel et al. 2015)	x	x	x	x
8	2	(Sahmel and Ramachandran 2022)	x	x	x	x
9	2	(Beamer et al. 2009a)	x	x	x	x
10	2	(Gorman Ng et al. 2013)	x	x	x	x
11	2	(Cohen Hubal et al. 2005)	x	x	x	x
12	3	(Kastury et al. 2018)	x	x	x	x
13	3	(Dartey et al. 2014)	x	x	x	x

Supplementary Table 3: Detailed search strings for the three topics.

No.	Topic	Search string
1	Mechanisms	(oral OR ingest* OR inadvertent) AND (expos* OR ingest*) AND (mechanism* OR factor* OR emerge* OR appear* OR develop*) NOT (pain OR surg* OR drug OR disease OR vaccine OR injury OR tooth OR teeth OR dent* OR disorder OR taste OR symptom OR DNA OR intravenous OR emergency OR diabetes OR cancer) NOT (fertil* OR newborne OR reproductive) NOT (bioaccess* OR excret* OR pharmaco*) NOT (food OR feed* OR diet*) NOT (educat* OR ecosytem OR animal)
2	Transfer efficiency	("transfer efficienc*" OR (dermal AND transfer)) AND (dermal OR hand* OR arm* OR oral OR peri-oral OR perioral) NOT (virus OR bacteria OR fomite*) NOT (heat)
3	Aerosols	("oral exposure" OR ingestion OR gastrointestinal) AND (aerosol OR (airborne AND particle) OR (lung AND clear*)) NOT (rats OR mice) NOT (transplant* OR surgery OR marine OR endoscopy)

Supplementary Table 4: Dates of last search for all search strategies, databases and websites. Simplified search strategies for websites.

Search strategy	Database / Website	Date of last search
1	Web of Science	21.08.2023
1	Pubmed	21.08.2023
mechanism oral exposure	IOM	22.08.2023
mechanism ingestion	HSE	22.08.2023
Mechanismen orale Exposition	BAuA	22.08.2023

(oral OR ingest* OR inadvertent) AND (expos* OR ingest*) AND (mechanism* OR factor* OR emerge* OR appear* OR develop*)	RIVM	22.08.2023
1	TNO	22.08.2023
(mechanism AND "oral exposure")	EPA	22.08.2023
oral AND exposure AND mechanism	NIOSH	22.08.2023
1	WHO	22.08.2023
1	OECD	22.08.2023
1	COCHRANE	22.08.2023
1	Deutsche Nationalbibliothek	22.08.2023
(oral OR ingest* OR inadvertent) AND (expos* OR ingest*) AND (mechanism* OR factor* OR emerge* OR appear* OR develop*)	bergischbib	22.08.2023
2	Web of Science	21.08.2023
2	Pubmed	21.08.2023
dermal transfer efficiency	IOM	22.08.2023
ingestion transfer	HSE	22.08.2023
dermal Transfer	BAuA	22.08.2023
("transfer efficienc*" OR (dermal AND transfer)) AND (dermal OR hand* OR arm* OR oral OR peri-oral OR perioral)	RIVM	22.08.2023
2	TNO	22.08.2023
transfer efficiency	EPA	22.08.2023
transfer AND efficiency	NIOSH	22.08.2023
2	WHO	22.08.2023
("transfer efficienc*" OR (dermal AND transfer)) AND (dermal OR hand* OR arm* OR oral OR peri-oral OR perioral)	OECD	22.08.2023
2	COCHRANE	22.08.2023
2	Deutsche Nationalbibliothek	22.08.2023
("transfer efficienc*" OR (dermal AND transfer)) AND (dermal OR hand* OR arm* OR oral OR peri-oral OR perioral)	bergischbib	22.08.2023
3	Web of Science	21.08.2023
3	Pubmed	21.08.2023
oral exposure aerosol	IOM	22.08.2023
ingestion aerosol	HSE	22.08.2023
orale Exposition Aerosol	BAuA	22.08.2023
orale Exposition Aerosol	RIVM	22.08.2023
("oral exposure" OR ingestion OR gastrointestinal) AND (aerosol OR (airborne AND particle) OR (lung AND clear*))	TNO	22.08.2023
("oral exposure" OR ingestion OR gastrointestinal) AND (aerosol OR (airborne AND particle) OR (lung AND clear*))	EPA	22.08.2023
oral exposure aerosol	NIOSH	22.08.2023
oral AND expsure AND aerosol	WHO	22.08.2023
3	OECD	22.08.2023

("oral exposure" OR ingestion OR gastrointestinal) AND (aerosol OR (airborne AND particle) OR (lung AND clear*))	COCHRANE	22.08.2023
3	Deutsche Nationalbibliothek	22.08.2023
("oral exposure" OR ingestion OR gastrointestinal) AND (aerosol OR (airborne AND particle) OR (lung AND clear*))	bergischbib	22.08.2023

Supplementary Table 5: Overview of all 175 included studies with extracted information for mechanisms, transfer and aerosols.

Study / source	First Author	Year	Substance group	Context	Children	Adult	Worker	Mechanisms	Transfer	Transfer: Quantitative	Aerosols
(Abbott and Maynard 2010)	Abbott	2010	Others	Consumer products				x	-	-	-
(Abrahams 2002)	Abrahams	2002	Soil / Sand	Outdoor	x	-	-	x	-	-	-
(Abuduwailil et al. 2015)	Abuduwailil	2015	Metals, Dust	Outdoor	x	x	-	x	-	-	-
(Ahmad et al. 2021)	Ahmad	2021	Biological substances	Waste water treatment plant (WWTP), reuse of water	-	x	-	x	-	-	-
(Ahmed et al. 2010)	Ahmed	2010	Biological substances	Waste water treatment plant (WWTP), reuse of water	-	x	-	x	-	-	-
(Akland et al. 2000)	Akland	2000	Pest control	Pest control	x	-	-	x	x	x	-
(Akpeimeh et al. 2021)	Akpeimeh	2021	Biological substances	Waste management	-	-	x	x	-	-	x
(Aleksandropoulou and Lazaridis 2013)	Aleksandropoulou	2013	PM	Outdoor, Indoor	-	x	-	-	-	-	x
(Al-Omran et al. 2021)	Al-Omran	2021	Flame retardants, Dust	Indoor	x	x	-	x	-	-	-
(Alpofead et al. 2017)	Alpofead	2017	PM	Others	-	x	-	-	-	-	x
(Amoah et al. 2022)	Amoah	2022	Biological substances	Waste water treatment plant (WWTP), reuse of water	-	-	x	x	-	-	-
(Api et al. 2007)	Api	2007	Organic compounds	Artificial situations	-	x	-	-	x	x	-
(Ayuso-Gabella et al. 2011)	Ayuso-Gabella	2011	Biological substances	Waste water treatment plant (WWTP), reuse of water	-	x	-	x	-	-	-
(Barraj et al. 2007)	Barraj	2007	Metals	Outdoor, Playground	x	-	-	x	x		-

(Beamer et al. 2009a)	Beamer	2009	Pest control	Pest control	-	x	-	x	x	x	-
(Beamer et al. 2009b)	Beamer	2009	Pest control	n.a.	x	-	-	x	x	x	-
(Benami et al. 2016)	Benami	2016	Biological substances	Waste water treatment plant (WWTP), reuse of water	-	-	-	x	-	-	x
(Bernard et al. 2008)	Bernard	2008	Pest control	Artificial situations	x	x	-	x	x	x	-
(Bernard et al. 2001)	Bernard	2001	Pest control	Outdoor	-	x	-	-	x	x	-
(Bernier and Vandenberg 2017)	Bernier	2017	Organic compounds	Consumer products	-	x	-	x	x	x	-
(Bertram et al. 2023)	Bertram	2023	Metals	Mining / industrial region	x	-	-	x	-	-	-
(Bolch et al. 2003)	Bolch	2003	Radioactive	Radioactivity	-	x	-	-	-	-	x
(Bowerbank et al. 2022)	Bowerbank	2022	Others	Outdoor	x	-	-	x	-	-	-
(Brouwer et al. 1999)	Brouwer	1999	Dust	Artificial situations	-	x	-	-	x	x	-
(Brown et al. 2013)	Brown	2013	Flame retardants, Dust	Indoor	x	x	-	x	-	-	-
(Brudecki et al. 2014)	Brudecki	2014	Radioactive	Radioactivity	x	x	-	-	-	-	x
(Caballero-Casero and Rubio 2022)	Caballero-Casero	2022	Others, Dust	Professional / industrial workplaces	-	-	x	x	-	-	-
(Chakraborty and Mondal 2018)	Chakraborty	2018	Metals	Indoor	x	-	-	x	-	-	-
(Chalvatzaki et al. 2014)	Chalvatzaki	2014	PM	Outdoor	-	-	x	-	-	-	x
(Chao et al. 2014)	Chao	2014	Flame retardants, Dust	Indoor	x	-	-	x	-	-	-
(Chatziprodromidou et al. 2022)	Chatziprodromidou	2022	Biological substances	Kindergarten / School / Playground	x	-	-	x	-	-	-
(Chen et al. 2021)	Chen	2021	Biological substances	Waste water treatment plant (WWTP), reuse of water	-	-	x	-	-	-	x
(Cherrie et al. 2006)	Cherrie	2006	Others, Pharmaceuticals / Drugs, Metals, Radioactive, Pest control	Professional / industrial workplaces	-	-	x	x	-	-	-
(Chowdhury et al. 2022)	Chowdhury	2022	Metals, PM	Waste management	-	x	-	x	-	-	-

(Clausen et al. 2016)	Clausen	2016	Organic compounds	Artificial situations	-	-	-	-	x	x	-
(Cohen Hubal et al. 2000)	Cohen Hubal	2000	Soil / Sand, Dust	Behaviour of children	x	-	-	x	-	-	-
(Cohen Hubal et al. 2005)	Cohen Hubal	2005	Others, Pest control	Artificial situations	x	x	-	x	x	x	-
(Cohen Hubal et al. 2006)	Cohen Hubal	2006	Pest control	Kindergarten / School / Playground	x	-	-	x	-	-	-
(Connor and Magee 2014)	Connor	2014	Metals	Professional / industrial workplaces	-	-	x	x	x	x	-
(Cushing et al. 2007)	Cushing	2007	Metals	Kindergarten / School / Playground	x	-	-	x	-	-	-
(da Silva et al. 2021)	da Silva	2021	Biological substances	Professional / industrial workplaces	x	x	-	x	-	-	-
(Damian 2011)	Damian	2011	Metals	Professional / industrial workplaces	-	-	x	x	x	x	-
(Dartey et al. 2014)	Dartey	2014	Metals	Professional / industrial workplaces	-	-	x	-	-	-	x
(Davis et al. 2021)	Davis	2021	Flame retardants, Organic compounds	Indoor	x	-	-	x	-	-	-
(de Bruin-Hoegée et al. 2020)	de Bruin-Hoegée	2020	Pharmaceuticals / Drug	Professional / industrial workplaces	-	-	x	-	x	x	-
(Dennehy 2000)	Dennehy	2000	Biological substances	Professional / industrial workplaces	x	-	-	x	-	-	-
(Driver et al. 2013)	Driver	2013	Pest control	Behaviour of children	x	-	-	x	x	-	-
(Dujardin et al. 2020)	Dujardin	2020	PM	Outdoor, Indoor	-	x	-	-	-	-	x
(English et al. 2017)	English	2017	Flame retardants	Indoor	x	-	-	x	-	-	-
(Eqani et al. 2016)	Eqani	2016	Metals, Dust	Outdoor	x	x	-	x	-	-	-
(Fan et al. 2015)	Fan	2015	Organic compounds	Artificial situations	-	x	-	-	x	-	-
(Fan et al. 2022)	Fan	2022	Flame retardants	Kindergarten / School / Playground	x	-	-	x	-	-	-
(Fantke et al. 2016)	Fantke	2016	n.a.	Consumer products	x	x	-	x	x	x	-
(Fatunsin et al. 2020)	Fatunsin	2020	Flame retardants	Consumer products	x	-	-	x	-	-	-

(Feng et al. 2023)	Feng	2023	Metals, Dust	Outdoor	x	-	-	x	-	-	-
(Ferguson et al. 2020)	Ferguson	2020	Soil / Sand	Outdoor	x	-	-	x	-	-	-
(Ferguson et al. 2009)	Ferguson	2009	Soil / Sand, Dust	Artificial situations	-	-	-	-	x	-	-
(Ferguson et al. 2008)	Ferguson	2008	Soil / Sand	Artificial situations	x	x	-	x	x	x	-
(Ferguson et al. 2012)	Ferguson	2012	Others	Artificial situations	-	x	-	-	x	-	-
(Garrido et al. 2019)	Garrido	2019	Organic compounds	Indoor	x	-	-	x	-	-	-
(Gloekler et al. 2021)	Gloekler	2021	Flame retardants	Artificial situations	x	-	-	x	x	x	-
(Goede et al. 2019)	Goede	2019	Liquids	Artificial situations	-	x	-	x	x	-	-
(Goix et al. 2016)	Goix	2016	Metals	Mining / industrial region	x	-	-	x	x	-	x
(Goncalves et al. 2021)	Goncalves	2021	(Waste) Water	Waste water treatment plant (WWTP), reuse of water	-	x	-	x	-	-	-
(Gorman Ng et al. 2016)	Gorman Ng	2016	n.a.	Professional / industrial workplaces	-	-	x	x	-	-	-
(Gorman Ng et al. 2012)	Gorman Ng	2012	n.a.	Professional / industrial workplaces	-	-	x	x	x	x	-
(Gorman Ng et al. 2013)	Gorman Ng	2013	Solids, Liquids	Artificial situations	-	x	-	-	x	x	-
(Gorman Ng et al. 2014)	Gorman Ng	2014	Solids, Liquids	n.a.	-	x	-	x	x	x	-
(Gorman Ng et al. 2017)	Gorman Ng	2017	Metals	Professional / industrial workplaces	-	-	x	x	-	-	-
(Gosselin et al. 2008)	Gosselin	2008	Pest control	Pest control	x	x	-	x	-	-	-
(Gray et al. 2005)	Gray	2005	(Waste) Water	Waste management	-	-	x	x	-	-	-
(Hamilton et al. 2017)	Hamilton	2017	Biological substances	Waste water treatment plant (WWTP), reuse of water	-	x	-	x	-	-	-
(Harrad et al. 2010)	Harrad	2010	Flame retardants, Organic compounds, Dust	Indoor	x	x	-	x	-	-	-
(Hemond and Solo-Gabriele 2004)	Hemond	2004	Metals	Kindergarten / School / Playground	x	-	-	x	x	x	-
(Hettiarachchi et al. 2022)	Hettiarachchi	2022	Dust	Outdoor, Indoor	-	x	-	-	-	-	x

(Heusinkveld et al. 2021)	Heusinkveld	2021	Metals	Mining / industrial region	x	-	-	x	-	-	-
(Hristozov et al. 2018)	Hristozov	2018	Pest control	Pest control	x	x	x	x	-	-	-
(Hsi et al. 2018)	Hsi	2018	Soil / Sand	Artificial situations	x	-	-	x	x	-	-
(Huang et al. 2022)	Huang	2022	Metals, Dust	Outdoor	x	x	-	x	-	-	-
(Huang et al. 2017)	Huang	2017	n.a.	Consumer products	x	x	-	x	x	x	-
(Hubal et al. 2008)	Hubal	2008	Others, Pest control	Artificial situations	-	x	-	x	x	x	-
(Hubbard et al. 2022)	Hubbard	2022	Soil / Sand, Dust	Outdoor, Indoor	-	x	x	x	x	-	-
(Hunt et al. 2008)	Hunt	2008	Metals, Dust	Artificial situations	x	-	-	x	x	-	-
(Ikegami et al. 2014)	Ikegami	2014	Metals, Soil / Sand	Kindergarten / School / Playground	x	-	-	x	x	-	-
(Irvine et al. 2014)	Irvine	2014	Soil / Sand	Outdoor	x	-	-	x	-	-	-
(James et al. 2012)	James	2012	Metals, Organic compounds	Outdoor	-	x	-	x	-	-	x
(Jiang et al. 2022)	Jiang	2022	Metals	Mining / industrial region	x	-	-	x	-	-	-
(Karwowski et al. 2017)	Karwowski	2017	Metals	Consumer products	x	-	-	x	x	-	x
(Kaur et al. 2020)	Kaur	2020	Metals	Outdoor	x	-	-	x	-	-	-
(Khokhryakov et al. 2005)	Khokhryakov	2005	Radioactive	Radioactivity	-	-	x	-	-	-	x
(Kumar and Scott Clark 2009)	Kumar	2009	Metals, Dust	Indoor	x	-	-	x	-	-	-
(Lacey et al. 2010)	Lacey	2010	Biological substances	Professional / industrial workplaces	-	-	x	x	-	-	-
(Li et al. 2020)	Li	2020	Metals, Dust	Outdoor	x	-	-	x	-	-	-
(Li et al. 2023)	Li	2023	Soil / Sand, Dust	Outdoor, Indoor	x	x	x	x	x	-	-
(Lin et al. 2015)	Lin	2015	Metals, Dust	Indoor	x	-	-	x	-	-	-
(Liu et al. 2014)	Liu	2014	Pest control	Pest control	x	-	-	x	-	-	-
(Ljung et al. 2006)	Ljung	2006	Soil / Sand, Metals	Kindergarten / School / Playground	x	-	-	x	-	-	-
(Ma et al. 2018)	Ma	2018	Organic compounds	Kindergarten / School / Playground	x	-	-	x	x	x	-
(Ma et al. 2022)	Ma	2022	Biological substances	Waste water treatment plant (WWTP), reuse of water	-	-	x	x	-	-	x

(Mackevica et al. 2018)	Mackevica	2018	Others	Consumer products	-	-	-	x	-	-	-
(Madrid et al. 2008)	Madrid	2008	Metals	Outdoor	x	-	-	x	-	-	-
(Mammi-Galani et al. 2017)	Mammi-Galani	2017	PM	Outdoor, Indoor	-	x	x	-	-	-	x
(Matsubara and Katayama 2019)	Matsubara	2019	Biological substances	Waste water treatment plant (WWTP), reuse of water	-	-	x	x	-	-	-
(Mbareche et al. 2022)	Mbareche	2022	Biological substances	Waste water treatment plant (WWTP), reuse of water	-	-	x	x	-	-	-
(Moor et al. 2021)	Moor	2021	Biological substances	Professional / industrial workplaces	-	-	x	x	-	-	x
(Moya and Phillips 2014)	Moya	2014	Soil / Sand, Dust	Outdoor, Indoor	x	-	-	x	x	-	-
(Ozkaynak et al. 2011)	Ozkaynak	2011	Soil / Sand, Dust	n.a.	x	-	-	x	x	x	-
(Özkaynak et al. 2022)	Özkaynak	2022	Soil / Sand, Dust	Outdoor, Indoor	x	-	-	x	-	-	-
(Pambianchi et al. 2022)	Pambianchi	2022	PM, Others	n.a.	-	x	-	x	-	-	x
(Pizzol et al. 2013)	Pizzol	2013	Metals	Waste management	x	-	-	x	-	-	-
(Platten et al. 2016)	Platten	2016	Metals	Artificial situations	x	-	-	x	x	x	-
(Poothong et al. 2019)	Poothong	2019	Organic compounds	Indoor	-	x	-	x	-	-	-
(Preece et al. 2021)	Preece	2021	Biological substances	Outdoor	x	-	-	x	-	-	-
(Rajaee et al. 2015)	Rajaee	2015	Metals	Mining / industrial region	x	x	x	x	-	-	-
(Ramwell et al. 2006)	Ramwell	2006	Pest control	Artificial situations	-	-	x	x	x	x	-
(Rauert et al. 2016)	Rauert	2016	Flame retardants	Artificial situations	-	-	-	-	x	-	-
(Rocha et al. 2021)	Rocha	2021	Pharmaceuticals / Drug	Professional / industrial workplaces	-	-	x	x	-	-	-
(Rodes et al. 2001)	Rodes	2001	Dust	Artificial situations	-	x	-	-	x	x	-
(Rohrer et al. 2003)	Rohrer	2003	Pest control	Artificial situations	x	-	-	x	x	x	-
(Roth 2006)	Roth	2006	Metals	Professional / industrial workplaces	-	-	x	-	-	-	x
(Rowbotham et al. 2000)	Rowbotham	2000	Metals	Outdoor	x	x	-	x	-	-	-
(Sahmel et al. 2022)	Sahmel	2022	Metals	Artificial situations	-	x	-	x	x	x	-

(Sahmel et al. 2015)	Sahmel	2015	Metals	Artificial situations	x	x	x	x	x	x	-
(Sahmel and Ramachandran 2022)	Sahmel	2022	Metals	Artificial situations	-	x	-	-	x	x	-
(Salocks et al. 2014)	Salocks	2014	Pharmaceuticals / Drug	Artificial situations	x	-	-	x	x	-	-
(Sankaran et al. 2015)	Sankaran	2015	Pest control	Pest control	-	-	x	-	x	x	-
(Schirmer 2010)	Schirmer	2010	Radioactive	Others, Radioactivity	-	-	x	x	x	x	-
(Schleier et al. 2009)	Schleier	2009	Pest control	Pest control	x	x	-	x	-	-	-
(Schoen et al. 2017)	Schoen	2017	Biological substances	Waste water treatment plant (WWTP), reuse of water	-	x	-	x	-	-	-
(Schoen et al. 2018)	Schoen	2018	Biological substances	Indoor	-	x	-	x	-	-	-
(Sen et al. 2002)	Sen	2002	Metals	Professional / industrial workplaces	-	-	x	x	-		-
(Shankar et al. 2017)	Shankar	2017	Others	Professional / industrial workplaces	-	-	x	x	-	-	-
(Shay et al. 2013)	Shay	2013	Metals	Professional / industrial workplaces	-	-	x	x	-	-	-
(Smith et al. 2014)	Smith	2014	Radioactive	n.a.	-	x	-	-	-	-	x
(Soltani et al. 2021)	Soltani	2021	Others	Indoor	x	-	-	x	-	-	-
(Stefaniak et al. 2021)	Stefaniak	2021	Powder / Particle / Fibre	Artificial situations	-	x	-	-	x	-	-
(Sturm 2007)	Sturm	2007	Powder / Particle / Fibre	n.a.	-	x	-	-	-	-	x
(Sturm and Hofmann 2006)	Sturm	2006	Powder / Particle / Fibre	n.a.	-	x	-	-	-	-	x
(Sturm and Hofmann 2009)	Sturm	2009	Powder / Particle / Fibre	n.a.	-	x	-	-	-	-	x
(Sugeng et al. 2017)	Sugeng	2017	Flame retardants	Indoor	x	-	-	x	-	-	-
(Swartjes and Janssen 2016)	Swartjes	2016	Metals	Outdoor	x	-	-	x	x	x	-
(Teunis et al. 2016)	Teunis	2016	Biological substances	Behaviour of children	x	-	-	x	-	-	-

(Tsakirakis et al. 2018)	Tsakirakis	2018	Pest control	Artificial situations	-	x	x	-	x	x	-
(Tsou et al. 2018)	Tsou	2018	Soil / Sand	Outdoor	x	-	-	x	x	-	-
(Tulve et al. 2011)	Tulve	2011	Pest control	Outdoor, Indoor	x	-	-	x	-	-	-
(Van Dyke et al. 2014)	Van Dyke	2014	Pharmaceuticals / Drug	Artificial situations	-	x	x	-	x	x	-
(Van Hooste et al. 2010)	Van Hooste	2010	Biological substances	Waste water treatment plant (WWTP), reuse of water	-	-	x	x	-	-	-
(Van Horne et al. 2023)	Van Horne	2023	Metals	Mining / industrial region	-	x	-	x	x	x	-
(Walker et al. 2022)	Walker	2022	Liquids	Artificial situations	-	x	-	-	x	x	-
(Wang et al. 2011)	Wang	2011	Soil / Sand, Organic compounds	Outdoor	x	-	-	x	-	-	-
(Wang et al. 2022)	Wang	2022	Liquids	Mining / industrial region	-	-	x	-	x	x	-
(Wang et al. 2021)	Wang	2021	Metals	Outdoor, Indoor	x	-	-	x	-	-	-
(Watson et al. 2018)	Watson	2018	(Waste) Water, Biological substances	Others	-	-	x	x	-	-	-
(Weeks et al. 2021)	Weeks	2021	Soil / Sand, Metals	Outdoor	x	x	-	x	-	-	-
(Weiss et al. 2018)	Weiss	2018	Plasticizer	Indoor	x	-	-	x	-	-	-
(Wensing et al. 2005)	Wensing	2005	Flame retardants, Plasticizer	Indoor	x	x	-	x	-	-	-
(Williams et al. 2008)	Williams	2008	Pest control	Indoor	-	-	-	x	x	x	-
(Wu et al. 2011)	Wu	2011	Others	Outdoor	-	x	-	x	-	-	-
(Wu et al. 2022)	Wu	2022	Organic compounds	Artificial situations	-	-	-	x	x	x	-
(Xie et al. 2016)	Xie	2016	Plasticizer	Consumer products	-	-	-	-	x	-	-
(Xu et al. 2016)	Xu	2016	Flame retardants	Indoor	-	x	-	x	-	-	-
(Xue et al. 2007)	Xue	2007	n.a.	Behaviour of children	x	-	-	x	-	-	-
(Xue et al. 2006)	Xue	2006	Metals	Kindergarten / School / Playground	x	-	-	x	x	x	-

(Yang et al. 2020)	Yang	2020	Flame retardants, Organic compounds	Indoor	x	-	-	x	-	-	-
(Yoshida-Ohuchi and Shinohara 2020)	Yoshida-Ohuchi	2020	Dust	Indoor	-	x	-	x	x	-	-
(Yusuf et al. 2023)	Yusuf	2023	Radioactive	Radioactivity	-	-	x	x	-	-	x
(Zartarian et al. 2006)	Zartarian	2006	Metals	Outdoor, Playground	x	-	-	x	-	-	-
(Zartarian et al. 2012)	Zartarian	2012	Pest control	Pest control	x	-	-	x	-	-	-
(Zhang et al. 2023)	Zhang	2023	Flame retardants, Plasticizer	Consumer products	x	-	-	x	-	-	-
(Zhang et al. 2022)	Zhang	2022	Organic compounds	Outdoor	x	-	-	x	-	-	-
(Zhao et al. 2021)	Zhao	2021	Organic compounds	Waste management	-	-	x	x	-	-	-
(Zhao et al. 2023a)	Zhao	2023	Organic compounds	Waste management	-	x	x	x	-	-	-
(Zhao et al. 2023b)	Zhao	2023	Organic compounds	Consumer products	x	-	-	x	-	-	-
(Zheng et al. 2022)	Zheng	2022	Metals, Dust	Kindergarten / School / Playground	x	-	-	x	-	-	-
(Zhong et al. 2020)	Zhong	2020	Metals	Outdoor	x	x	-	x	-	-	-

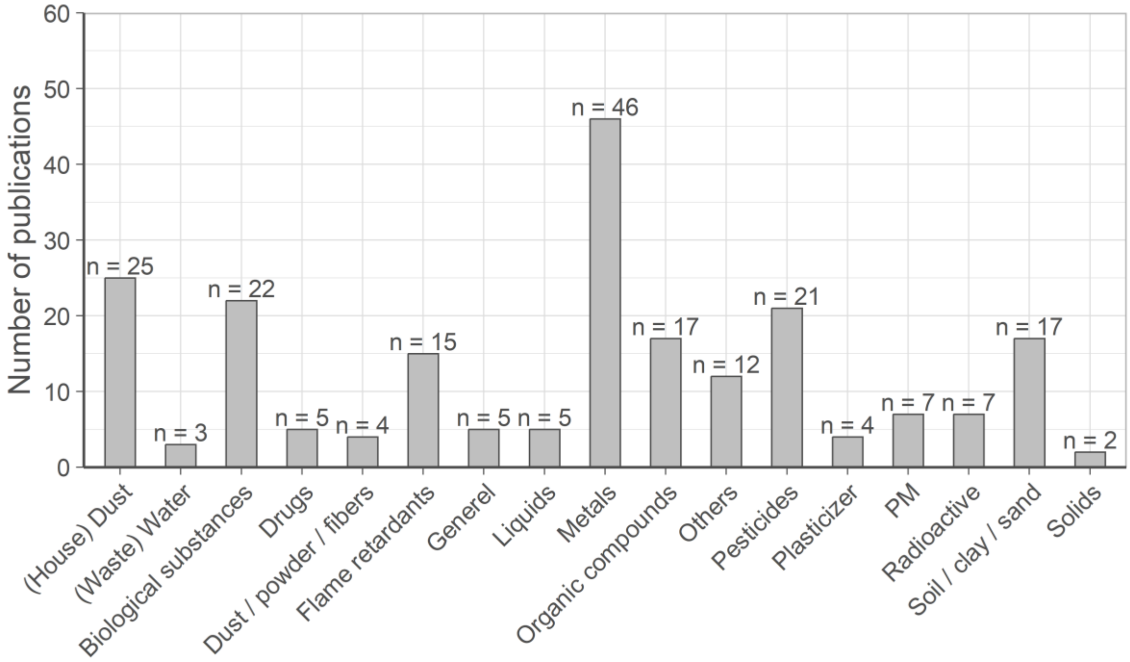

Supplementary Figure 1: Substance groups which were investigated in the included studies.

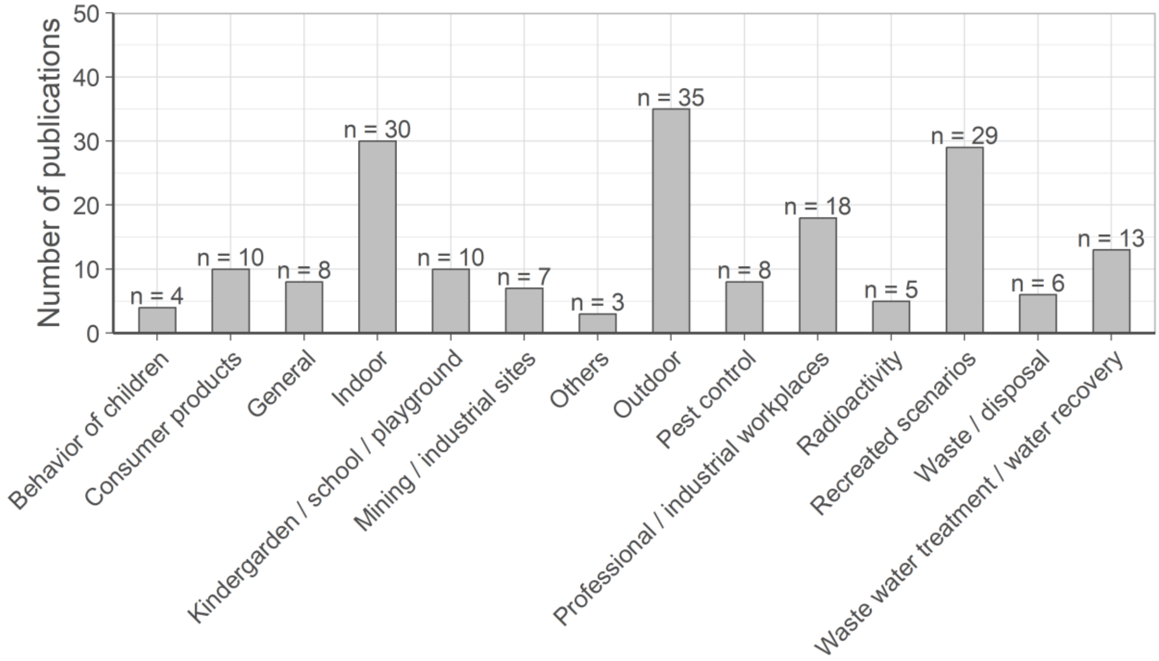

Supplementary Figure 2: Context which is investigated in included studies.

Literature used for search evaluation or identified in systematic search

Abbott LC, Maynard AD. 2010. Exposure assessment approaches for engineered nanomaterials. Risk Analysis. 30(11):1634-1644.

Abrahams PW. 2002. Soils: Their implications to human health. Sci Total Environ. 291(1-3):1-32.

Abuduwailil J, Zhaoyong Z, Fengqing J. 2015. Evaluation of the pollution and human health risks posed by heavy metals in the atmospheric dust in ebinur basin in northwest china. Environ Sci Pollut Res Int. 22(18):14018-14031.

Ahmad J, Ahmad M, Usman ARA, Al-Wabel MI. 2021. Prevalence of human pathogenic viruses in wastewater: A potential transmission risk as well as an effective tool for early outbreak detection for covid-19. J Environ Manage. 298:113486.

Ahmed W, Vieritz A, Goonetilleke A, Gardner T. 2010. Health risk from the use of roof-harvested rainwater in southeast queensland, australia, as potable or nonpotable water, determined using quantitative microbial risk assessment. Appl Environ Microbiol. 76(22):7382-7391.

Akland GG, Pellizzari ED, Hu Y, Roberds M, Rohrer CA, Leckie JO, Berry MR. 2000. Factors influencing total dietary exposures of young children. J Expo Anal Environ Epidemiol. 10(6 Pt 2):710-722.

Akpeimeh GF, Fletcher LA, Evans BE, Ibanga IE. 2021. Quantitative microbial risk assessment (qmra) of workers exposure to bioaerosols at msw open dumpsites. Risk Anal. 41(10):1911-1924.

Al-Omran LS, Harrad S, Abou-Elwafa Abdallah M. 2021. A meta-analysis of factors influencing concentrations of brominated flame retardants and organophosphate esters in indoor dust. Environ Pollut. 285:117262.

Aleksandropoulou V, Lazaridis M. 2013. Development and application of a model (exdom) for calculating the respiratory tract dose and retention of particles under variable exposure conditions. Air Quality Atmosphere and Health. 6(1):13-26.

Alpofead JAH, Davidson CM, Littlejohn D. 2017. A novel two-step sequential bioaccessibility test for potentially toxic elements in inhaled particulate matter transported into the gastrointestinal tract by mucociliary clearance. Analytical and Bioanalytical Chemistry. 409(12):3165-3174.

Amoah ID, Kumari S, Bux F. 2022. A probabilistic assessment of microbial infection risks due to occupational exposure to wastewater in a conventional activated sludge wastewater treatment plant. Sci Total Environ. 843:156849.

Api AM, Bredbenner A, McGowen M, Niemiera D, Parker L, Renskers K, Selim S, Sgaramella R, Signorelli R, Tedrow S et al. 2007. Skin contact transfer of three fragrance residues from candles to human hands. Regul Toxicol Pharmacol. 48(3):279-283.

Ayuso-Gabella N, Page D, Masciopinto C, Aharoni A, Salgot M, Wintgens T. 2011. Quantifying the effect of managed aquifer recharge on the microbiological human health risks of irrigating crops with recycled water. Agricultural Water Management. 99(1):93-102.

Barraj LM, Tsuji JS, Scrafford CG. 2007. The sheds-wood model: Incorporation of observational data to estimate exposure to arsenic for children playing on cca-treated wood structures. Environ Health Perspect. 115(5):781-786.

Beamer P, Canales RA, Leckie JO. 2009a. Developing probability distributions for transfer efficiencies for dermal exposure. J Expo Sci Environ Epidemiol. 19(3):274-283.

Beamer PI, Canales RA, Bradman A, Leckie JO. 2009b. Farmworker children's residential non-dietary exposure estimates from micro-level activity time series. Environment International. 35(8):1202-1209.

Benami M, Busgang A, Gillor O, Gross A. 2016. Quantification and risks associated with bacterial aerosols near domestic greywater-treatment systems. Sci Total Environ. 562:344-352.

Bernard CE, Berry MR, Wymer LJ, Melnyk LJ. 2008. Sampling household surfaces for pesticide residues: Comparison between a press sampler and solvent-moistened wipes. Science of the Total Environment. 389(2-3):514-521.

Bernard CE, Nuygen H, Truong D, Krieger RI. 2001. Environmental residues and biomonitoring estimates of human insecticide exposure from treated residential turf. Archives of Environmental Contamination and Toxicology. 41(2):237-240.

Bernier MR, Vandenberg LN. 2017. Handling of thermal paper: Implications for dermal exposure to bisphenol a and its alternatives. PLoS One. 12(6):e0178449.

Bertram J, Ramolla C, Esser A, Schettgen T, Fohn N, Steib J, Kraus T. 2023. Blood lead monitoring in a former mining area in euskirchen, germany: Results of a representative random sample in 3- to 17-year-old children and minors. Environ Sci Pollut Res Int. 30(8):20995-21009.

Bolch WE, Huston TE, Farfán EB, Vernetson WG, Bolch WE. 2003. Influences of parameter uncertainties within the icrp-66 respiratory tract model: Particle clearance. Health Phys. 84(4):421-435.

Bowerbank SL, Gallidabino MD, Dean JR. 2022. Plant poisons in the garden: A human risk assessment. Separations. 9(10).

Brouwer DH, Kroese R, Van Hemmen JJ. 1999. Transfer of contaminants from surface to hands: Experimental assessment of linearity of the exposure process, adherence to the skin, and area exposed during fixed pressure and repeated contact with surfaces contaminated with a powder. Appl Occup Environ Hyg. 14(4):231-239.

Brown JS, Gordon T, Price O, Asgharian B. 2013. Thoracic and respirable particle definitions for human health risk assessment. Part Fibre Toxicol. 10:12.

Brudecki K, Li WB, Meisenberg O, Tschiersch J, Hoeschen C, Oeh U. 2014. Age-dependent inhalation doses to members of the public from indoor short-lived radon progeny. Radiat Environ Biophys. 53(3):535-549.

Caballero-Casero N, Rubio S. 2022. Identification of bisphenols and derivatives in greenhouse dust as a potential source for human occupational exposure. Anal Bioanal Chem. 414(18):5397-5409.

Chakraborty D, Mondal NK. 2018. Assessment of health risk of children from traditional biomass burning in rural households. Exposure and Health. 10(1):15-26.

Chalvatzaki E, Aleksandropoulou V, Lazaridis M. 2014. A case study of landfill workers exposure and dose to particulate matter-bound metals. Water Air and Soil Pollution. 225(1).
Chao HR, Shy CG, Huang HL, Koh TW, Tok TS, Chen SCC, Chiang BA, Kuo YM, Chen KC, Chang-Chien GP. 2014. Particle-size dust concentrations of polybrominated diphenyl ethers (pbdes) in southern taiwanese houses and assessment of the pbde daily intakes in toddlers and adults. Aerosol and Air Quality Research. 14(4):1299-1309.
Chatziprodromidou IP, Chatziantoniou S, Vantarakis G, Vantarakis A. 2022. Risk factor analysis of children's exposure to microbial pathogens in playgrounds. Risk Anal. 42(2):334-343.
Chen YH, Yan C, Yang YF, Ma JX. 2021. Quantitative microbial risk assessment and sensitivity analysis for workers exposed to pathogenic bacterial bioaerosols under various aeration modes in two wastewater treatment plants. Sci Total Environ. 755(Pt 2):142615.
Cherrie JW, Semple S, Christopher Y, Saleem A, Hughson GW, Philips A. 2006. How important is inadvertent ingestion of hazardous substances at work? Ann Occup Hyg. 50(7):693-704.
Chowdhury M, Ghosh S, Padhy PK. 2022. Effects of indoor air pollution on tribal community in rural india and health risk assessment due to domestic biomass burning: A realistic approach using the lung deposition model. Environ Sci Pollut Res Int. 29(39):59606-59618.
Clausen PA, Spaan S, Brouwer DH, Marquart H, le Feber M, Engel R, Geerts L, Jensen KA, Kofoed-Sørensen V, Hansen B et al. 2016. Experimental estimation of migration and transfer of organic substances from consumer articles to cotton wipes: Evaluation of underlying mechanisms. J Expo Sci Environ Epidemiol. 26(1):104-112.
Cohen Hubal EA, Egeghy PP, Leovic KW, Akland GG. 2006. Measuring potential dermal transfer of a pesticide to children in a child care center. Environ Health Perspect. 114(2):264-269.
Cohen Hubal EA, Sheldon LS, Burke JM, McCurdy TR, Berry MR, Rigas ML, Zartarian VG, Freeman NC. 2000. Children's exposure assessment: A review of factors influencing children's exposure, and the data available to characterize and assess that exposure. Environ Health Perspect. 108(6):475-486.
Cohen Hubal EA, Suggs JC, Nishioka MG, Ivancic WA. 2005. Characterizing residue transfer efficiencies using a fluorescent imaging technique. J Expo Anal Environ Epidemiol. 15(3):261-270.
Connor K, Magee B. 2014. A quantitative assessment of risks of heavy metal residues in laundered shop towels and their use by workers. Regul Toxicol Pharmacol. 70(1):125-137.
Cushing CA, Golden R, Lowney YW, Holm SE. 2007. Human health risk evaluation of acq-treated wood. Human and Ecological Risk Assessment. 13(5):1014-1041.
da Silva FAF, de Brito BB, Santos MLC, Marques HS, da Silva Júnior RT, de Carvalho LS, de Sousa Cruz S, Rocha GR, Correa Santos GL, de Souza KC et al. 2021. Transmission of severe acute respiratory syndrome coronavirus 2 via fecal-oral: Current knowledge. World J Clin Cases. 9(28):8280-8294.
Damian P. 2011. Development of a health risk-based surface contamination cleanup standard for occupational exposure to beryllium. Toxicol Mech Methods. 21(2):97-102.
Dartey E, Berlinger B, Thomassen Y, Ellingsen DG, Odland J, Nartey VK, Yeboah FA, Weinbruch S. 2014. Bioaccessibility of lead in airborne particulates from car battery repair work. Environ Sci Process Impacts. 16(12):2782-2788.
Davis A, Ryan PB, Cohen JA, Harris D, Black M. 2021. Chemical exposures from upholstered furniture with various flame retardant technologies. Indoor Air. 31(5):1473-1483.
de Bruin-Hoegée M, de Koning MC, Cochrane L, Joosen MJA. 2020. Contact transfer risk from fentanyl-contaminated rsdl® kit. Toxicol Lett. 319:237-241.
Dennehy PH. 2000. Transmission of rotavirus and other enteric pathogens in the home. Pediatr Infect Dis J. 19(10 Suppl):S103-105.
Deubner DC, Lowney YW, Paustenbach DJ, Warmerdam J. 2001. Contribution of incidental exposure pathways to total beryllium exposures. Appl Occup Environ Hyg. 16(5):568-578.
Driver J, Ross J, Pandian M, Assaf N, Osimitz T, Holden L. 2013. Evaluation of predictive algorithms used for estimating potential postapplication, nondietary ingestion exposures to pesticides associated with children's hand-to-mouth behavior. J Toxicol Environ Health A. 76(9):556-586.
Dujardin CE, Mars RAT, Manemann SM, Kashyap PC, Clements NS, Hassett LC, Roger VL. 2020. Impact of air quality on the gastrointestinal microbiome: A review. Environmental Research. 186.
English K, Chen Y, Toms LM, Jagals P, Ware RS, Mueller JF, Sly PD. 2017. Polybrominated diphenyl ether flame retardant concentrations in faeces from young children in queensland, australia and associations with environmental and behavioural factors. Environ Res. 158:669-676.
Eqani S, Khalid R, Bostan N, Saqib Z, Mohmand J, Rehan M, Ali N, Katsoyiannis IA, Shen H. 2016. Human lead (pb) exposure via dust from different land use settings of pakistan: A case study from two urban mountainous cities. Chemosphere. 155:259-265.
Fan R, Zeng B, Liu X, Chen C, Zhuang Q, Wang Y, Hu M, Lv Y, Li J, Zhou Y et al. 2015. Levels of bisphenol-a in different paper products in guangzhou, china, and assessment of human exposure via dermal contact. Environ Sci Process Impacts. 17(3):667-673.
Fan Y, Chen Q, Wang Z, Zhang X, Zhao J, Huang X, Wei P, Hu P, Cao Z. 2022. Identifying dermal exposure as the dominant pathway of children's exposure to flame retardants in kindergartens. Sci Total Environ. 808:152004.
Fantke P, Ernstoff AS, Huang L, Csiszar SA, Jolliet O. 2016. Coupled near-field and far-field exposure assessment framework for chemicals in consumer products. Environment International. 94:508-518.
Fatunsin OT, Oluseyi TO, Drage D, Abdallah MA, Turner A, Harrad S. 2020. Children's exposure to hazardous brominated flame retardants in plastic toys. Sci Total Environ. 720:137623.

Feng D, Gong P, Li Y, Li N, Dong Z, Zhu Z, Jiang R, Deng S. 2023. Risk assessment and source apportionment of heavy metals pollution from atmospheric deposition in nanjing, china. Heliyon. 9(8):e18858.
Ferguson A, Bursac Z, Coleman S, Johnson W. 2009. Comparisons of computer-controlled chamber measurements for soil-skin adherence from aluminum and carpet surfaces. Environ Res. 109(3):207-214.
Ferguson A, Bursac Z, Johnson W, Davis J. 2012. Computer controlled chamber measurements for clay adherence relevant for potential dioxin exposure through skin. J Environ Sci Health A Tox Hazard Subst Environ Eng. 47(3):382-388.
Ferguson A, Kumar Dwivedi A, Ehindero E, Adelabu F, Rattler K, Perone HR, Montas L, Mena K, Solo-Gabriele H. 2020. Soil, hand, and body adherence measures across four beach areas: Potential influence on exposure to oil spill chemicals. Int J Environ Res Public Health. 17(12).
Ferguson AC, Bursac Z, Biddle D, Coleman S, Johnson W. 2008. Soil-skin adherence from carpet: Use of a mechanical chamber to control contact parameters. J Environ Sci Health A Tox Hazard Subst Environ Eng. 43(12):1451-1458.
Garrido JA, Parthasarathy S, Moschet C, Young TM, McKone TE, Bennett DH. 2019. Exposure assessment for air-to-skin uptake of semivolatile organic compounds (svocs) indoors. Environ Sci Technol. 53(3):1608-1616.
Gloekler LE, Barlow CA, Tvermoes B, La Guardia MJ, Sahmel J. 2021. A pilot study to characterize hand-to-mouth transfer efficiency of organophosphate flame retardants identified in infant products. Human and Ecological Risk Assessment. 27(9-10):2288-2310.
Goede HA, McNally K, Gorce JP, Marquart H, Warren ND, Fransman W, Tischer M, Schinkel J. 2019. Dermal advanced reach tool (dart)-development of a dermal exposure model for low-volatile liquids. Ann Work Expo Health. 63(6):624-636.
Goix S, Uzu G, Oliva P, Barraza F, Calas A, Castet S, Point D, Masbou J, Duprey JL, Huayta C et al. 2016. Metal concentration and bioaccessibility in different particle sizes of dust and aerosols to refine metal exposure assessment. J Hazard Mater. 317:552-562.
Goncalves RF, Vaz LD, Peres M, Merlo SS. 2021. Microbiological risk from non-potable reuse of greywater treated by anaerobic filters associated to vertical constructed wetlands. Journal of Water Process Engineering. 39.
Gorman Ng M, Davis A, van Tongeren M, Cowie H, Semple S. 2016. Inadvertent ingestion exposure: Hand- and object-to-mouth behavior among workers. J Expo Sci Environ Epidemiol. 26(1):9-16.
Gorman Ng M, de Poot S, Schmid K, Cowie H, Semple S, van Tongeren M. 2013. Properties of liquids and dusts: How do they influence dermal loading during immersion, deposition, and surface contact exposure pathways? Ann Occup Hyg. 57(5):627-639.
Gorman Ng M, MacCalman L, Semple S, van Tongeren M. 2017. Field measurements of inadvertent ingestion exposure to metals. Annals of Work Exposures and Health. 61(9):1097-1107.
Gorman Ng M, Semple S, Cherrie JW, Christopher Y, Northage C, Tielemans E, Veroughstraete V, Van Tongeren M. 2012. The relationship between inadvertent ingestion and dermal exposure pathways: A new integrated conceptual model and a database of dermal and oral transfer efficiencies. Ann Occup Hyg. 56(9):1000-1012.
Gorman Ng M, van Tongeren M, Semple S. 2014. Simulated transfer of liquids and powders from hands and clothing to the mouth. J Occup Environ Hyg. 11(10):633-644.
Gosselin N, Valcke M, Belleville D, Samuel O. 2008. Human exposure to malathion during a possible vector-control intervention against west nile virus. I: Methodological framework for exposure assessment. Human and Ecological Risk Assessment. 14(6):1118-1137.
Gray D, Pollard SJ, Spence L, Smith R, Gronow JR. 2005. Spray irrigation of landfill leachate: Estimating potential exposures to workers and bystanders using a modified air box model and generalised source term. Environ Pollut. 133(3):587-599.
Hamilton KA, Ahmed W, Toze S, Haas CN. 2017. Human health risks for legionella and mycobacterium avium complex (mac) from potable and non-potable uses of roof-harvested rainwater. Water Res. 119:288-303.
Harrad S, de Wit CA, Abdallah MA, Bergh C, Björklund JA, Covaci A, Darnerud PO, de Boer J, Diamond M, Huber S et al. 2010. Indoor contamination with hexabromocyclododecanes, polybrominated diphenyl ethers, and perfluoroalkyl compounds: An important exposure pathway for people? Environ Sci Technol. 44(9):3221-3231.
Hemond HF, Solo-Gabriele HM. 2004. Children's exposure to arsenic from cca-treated wooden decks and playground structures. Risk Analysis. 24(1):51-64.
Hettiarachchi E, Das M, Cadol D, Frey BA, Rubasinghege G. 2022. The fate of inhaled uranium-containing particles upon clearance to gastrointestinal tract. Environmental Science-Processes & Impacts. 24(8):1257-1266.
Heusinkveld D, Ramirez-Andreotta MD, Rodríguez-Chávez T, Sáez AE, Betterton E, Rine K. 2021. Assessing children's lead exposure in an active mining community using the integrated exposure uptake biokinetic model. Expo Health. 13(3):517-533.
Hristozov D, Pizzol L, Basei G, Zabeo A, Mackevica A, Hansen SF, Gosens I, Cassee FR, de Jong W, Koivisto AJ et al. 2018. Quantitative human health risk assessment along the lifecycle of nano-scale copper-based wood preservatives. Nanotoxicology. 12(7):747-765.
Hsi HC, Hu CY, Tsou MC, Hu HJ, Özkaynak H, Bradham K, Hseu ZY, Dang W, Chien LC. 2018. Determination of hand soil loading, soil transfer, and particle size variations after hand-pressing and hand-mouthing activities. Sci Total Environ. 627:844-851.
Huang F, Liu B, Yu Y, Lv L, Luo X, Yin F. 2022. Heavy metals in road dust across china: Occurrence, sources and health risk assessment. Bull Environ Contam Toxicol. 109(2):323-331.
Huang L, Ernstoff A, Fantke P, Csiszar SA, Jolliet O. 2017. A review of models for near-field exposure pathways of chemicals in consumer products. Sci Total Environ. 574:1182-1208.

Hubal EA, Nishioka MG, Ivancic WA, Morara M, Egeghy PP. 2008. Comparing surface residue transfer efficiencies to hands using polar and nonpolar fluorescent tracers. Environ Sci Technol. 42(3):934-939.
Hubbard H, Özkaynak H, Glen G, Cohen J, Thomas K, Phillips L, Tulve N. 2022. Model-based predictions of soil and dust ingestion rates for u.S. Adults using the stochastic human exposure and dose simulation soil and dust model. Sci Total Environ. 846:157501.
Hunt A, Johnson DL, Brooks J, Griffith DA. 2008. Risk remaining from fine particle contaminants after vacuum cleaning of hard floor surfaces. Environ Geochem Health. 30(6):597-611.
Ikegami M, Yoneda M, Tsuji T, Bannai O, Morisawa S. 2014. Effect of particle size on risk assessment of direct soil ingestion and metals adhered to children's hands at playgrounds. Risk Anal. 34(9):1677-1687.
Irvine G, Doyle JR, White PA, Blais JM. 2014. Soil ingestion rate determination in a rural population of alberta, canada practicing a wilderness lifestyle. Science of the Total Environment. 470:138-146.
James K, Farrell RE, Siciliano SD. 2012. Comparison of human exposure pathways in an urban brownfield: Reduced risk from paving roads. Environ Toxicol Chem. 31(10):2423-2430.
Jiang Y, Wen H, Zhang Q, Yuan L, Liu L. 2022. Source apportionment and health risk assessment of potentially toxic elements in soil from mining areas in northwestern china. Environ Geochem Health. 44(5):1551-1566.
Karwowski MP, Morman SA, Plumlee GS, Law T, Kellogg M, Woolf AD. 2017. Toxicants in folk remedies: Implications of elevated blood lead in an american-born infant due to imported diaper powder. Environ Geochem Health. 39(5):1133-1143.
Kastury F, Smith E, Karna RR, Scheckel KG, Juhasz AL. 2018. An inhalation-ingestion bioaccessibility assay (iiba) for the assessment of exposure to metal(loid)s in pm10. Science of The Total Environment. 631-632:92-104.
Kaur M, Kumar A, Mehra R, Kaur I. 2020. Quantitative assessment of exposure of heavy metals in groundwater and soil on human health in reasi district, jammu and kashmir. Environ Geochem Health. 42(1):77-94.
Khokhryakov VF, Suslova KG, Vostrotin VV, Romanov SA, Eckerman KF, Krahenbuhl MP, Miller SC. 2005. Adaptation of the icrp publication 66 respiratory tract model to data on plutonium biokinetics for mayak workers. Health Phys. 88(2):125-132.
Kumar A, Scott Clark C. 2009. Lead loadings in household dust in delhi, india. Indoor Air. 19(5):414-420.
Lacey SE, Abelmann A, Dorevitch S. 2010. Exposure to human waste from spills while servicing aircraft lavatories: Hazards and methods of prevention. Ind Health. 48(1):123-128.
Li N, Zhang J, Yu H, Xu MH, Feng Q, Zhang JY, Wang XY, Wei PK, Fan YJ, Yan GX et al. 2023. A systematic characterization of soil/dust ingestion for typical subpopulations in china. Environmental Geochemistry and Health. 45(8):6199-6214.
Li Z, Liang T, Li K, Wang P. 2020. Exposure of children to light rare earth elements through ingestion of various size fractions of road dust in rees mining areas. Sci Total Environ. 743:140432.
Lin Y, Fang F, Wang F, Xu M. 2015. Pollution distribution and health risk assessment of heavy metals in indoor dust in anhui rural, china. Environ Monit Assess. 187(9):565.
Liu P, Wu CH, Chang XL, Qi XJ, Zheng ML, Zhou ZJ. 2014. Assessment of chlorpyrifos exposure and absorbed daily doses among infants living in an agricultural area of the province of jiangsu, china. Int Arch Occup Environ Health. 87(7):753-762.
Ljung K, Selinus O, Otabbong E, Berglund M. 2006. Metal and arsenic distribution in soil particle sizes relevant to soil ingestion by children. Applied Geochemistry. 21(9):1613-1624.
Ma J, An D, Cui B, Liu M, Zhu H, Li M, Ai X, Ali W, Yan C. 2022. What are the disease burden and its sensitivity analysis of workers exposing to staphylococcus aureus bioaerosol during warm and cold periods in a wastewater treatment plant? Environ Sci Pollut Res Int. 29(55):82938-82947.
Ma J, Pan LB, Wang Q, Lin CY, Duan XL, Hou H. 2018. Estimation of the daily soil/dust (sd) ingestion rate of children from gansu province, china via hand-to-mouth contact using tracer elements. Environ Geochem Health. 40(1):295-301.
Mackevica A, Olsson ME, Mines PD, Heggelund LR, Hansen SF. 2018. Dermal transfer quantification of nanoparticles from nano-enabled surfaces. Nanoimpact. 11:109-118.
Madrid F, Biasioli M, Ajmone-Marsan F. 2008. Availability and bioaccessibility of metals in fine particles of some urban soils. Arch Environ Contam Toxicol. 55(1):21-32.
Mammi-Galani E, Eleftheriadis K, Mendes L, Lazaridis M. 2017. Exposure and dose to particulate matter inside the subway system of athens, greece. Air Quality Atmosphere and Health. 10(8):1015-1028.
Matsubara K, Katayama H. 2019. Development of a portable detection method for enteric viruses from ambient air and its application to a wastewater treatment plant. Pathogens. 8(3).
Mbareche H, Dion-Dupont V, Veillette M, Brisebois E, Lavoie J, Duchaine C. 2022. Influence of seasons and sites on bioaerosols in indoor wastewater treatment plants and proposal for air quality indicators. J Air Waste Manag Assoc. 72(9):1000-1011.
Moor J, Wüthrich T, Aebi S, Mostacci N, Overesch G, Oppliger A, Hilty M. 2021. Influence of pig farming on human gut microbiota: Role of airborne microbial communities. Gut Microbes. 13(1):1-13.
Moya J, Phillips L. 2014. A review of soil and dust ingestion studies for children. J Expo Sci Environ Epidemiol. 24(6):545-554.
Özkaynak H, Glen G, Cohen J, Hubbard H, Thomas K, Phillips L, Tulve N. 2022. Model based prediction of age-specific soil and dust ingestion rates for children. J Expo Sci Environ Epidemiol. 32(3):472-480.
Ozkaynak H, Xue JP, Zartarian VG, Glen G, Smith L. 2011. Modeled estimates of soil and dust ingestion rates for children. Risk Analysis. 31(4):592-608.
Pambianchi E, Pecorelli A, Valacchi G. 2022. Gastrointestinal tissue as a "new" target of pollution exposure. IUBMB Life. 74(1):62-73.

Pizzol M, Moller F, Thomsen M. 2013. External costs of atmospheric lead emissions from a waste-to-energy plant: A follow-up assessment of indirect exposure via topsoil ingestion. Journal of Environmental Management. 121:170-178.
Platten WE, 3rd, Sylvest N, Warren C, Arambewela M, Harmon S, Bradham K, Rogers K, Thomas T, Luxton TP. 2016. Estimating dermal transfer of copper particles from the surfaces of pressure-treated lumber and implications for exposure. Sci Total Environ. 548-549:441-449.
Poothong S, Padilla-Sánchez JA, Papadopoulou E, Giovanoulis G, Thomsen C, Haug LS. 2019. Hand wipes: A useful tool for assessing human exposure to poly- and perfluoroalkyl substances (pfass) through hand-to-mouth and dermal contacts. Environ Sci Technol. 53(4):1985-1993.
Preece EP, Hobbs W, Hardy FJ, O'Garro L, Frame E, Sweeney F. 2021. Prevalence and persistence of microcystin in shoreline lake sediments and porewater, and associated potential for human health risk. Chemosphere. 272:129581.
Rajaee M, Long RN, Renne EP, Basu N. 2015. Mercury exposure assessment and spatial distribution in a ghanaian small-scale gold mining community. International Journal of Environmental Research and Public Health. 12(9):10755-10782.
Ramwell CT, Johnson PD, Corns H. 2006. Transferability of six pesticides from agricultural sprayer surfaces. Annals of Occupational Hygiene. 50(3):323-329.
Rauert C, Kuribara I, Kataoka T, Wada T, Kajiwara N, Suzuki G, Takigami H, Harrad S. 2016. Direct contact between dust and hbcd-treated fabrics is an important pathway of source-to-dust transfer. Sci Total Environ. 545-546:77-83.
Rocha SD, Gomes ANH, Zen PRG, Bica CG. 2021. Handling of antineoplastic drugs: A health concern among health care workers. Rev Bras Med Trab. 18(4):407-414.
Rodes CE, Newsome JR, Vanderpool RW, Antley JT, Lewis RG. 2001. Experimental methodologies and preliminary transfer factor data for estimation of dermal exposures to particles. J Expo Anal Environ Epidemiol. 11(2):123-139.
Rohrer CA, Hieber TE, Melnyk LJ, Berry MR. 2003. Transfer efficiencies of pesticides from household flooring surfaces to foods. J Expo Anal Environ Epidemiol. 13(6):454-464.
Roth JA. 2006. Homeostatic and toxic mechanisms regulating manganese uptake, retention, and elimination. Biol Res. 39(1):45-57.
Rowbotham AL, Levy LS, Shuker LK. 2000. Chromium in the environment: An evaluation of exposure of the uk general population and possible adverse health effects. J Toxicol Environ Health B Crit Rev. 3(3):145-178.
Sahmel J, Arnold S, Ramachandran G. 2022. Influence of repeated contacts on the transfer of elemental metallic lead between compartments in an integrated conceptual model for dermal exposure assessment. Journal of Toxicology and Environmental Health-Part a-Current Issues. 85(3):89-109.
Sahmel J, Hsu EI, Avens HJ, Beckett EM, Devlin KD. 2015. Estimation of hand-to-mouth transfer efficiency of lead. Ann Occup Hyg. 59(2):210-220.
Sahmel J, Ramachandran G. 2022. Potential influence of skin hydration and transepidermal water loss on the dermal transfer and loading of elemental metallic lead. Ann Work Expo Health. 66(7):923-936.
Salocks CB, Hui X, Lamel S, Hafeez F, Qiao P, Sanborn JR, Maibach HI. 2014. Dermal exposure to methamphetamine hydrochloride contaminated residential surfaces ii. Skin surface contact and dermal transfer relationship. Food Chem Toxicol. 66:1-6.
Sankaran G, Lopez T, Ries S, Ross J, Vega H, Eastmond DA, Krieger RI. 2015. Latex rubber gloves as a sampling dosimeter using a novel surrogate sampling device. Journal of Toxicology and Environmental Health-Part a-Current Issues. 78(17):1094-1104.
Schirmer A. 2010. Ingestion of 226ra from activated paints on military equipment: Transfer factors and doses. Health Phys. 99(4):568-571.
Schleier JJ, Macedo PA, Davis RS, Shama LM, Peterson RKD. 2009. A two-dimensional probabilistic acute human-health risk assessment of insecticide exposure after adult mosquito management. Stochastic Environmental Research and Risk Assessment. 23(5):555-563.
Schoen ME, Ashbolt NJ, Jahne MA, Garland J. 2017. Risk-based enteric pathogen reduction targets for non-potable and direct potable use of roof runoff, stormwater, and greywater. Microbial Risk Analysis. 5:32-43.
Schoen ME, Jahne MA, Garland J. 2018. Human health impact of non-potable reuse of distributed wastewater and greywater treated by membrane bioreactors. Microbial Risk Analysis. 9:72-81.
Sen D, Wolfson H, Dilworth M. 2002. Lead exposure in scaffolders during refurbishment construction activity--an observational study. Occup Med (Lond). 52(1):49-54.
Shankar K, Fung V, Seneviratne M, O'Donnell GE. 2017. Exposure to 4,4'-methylene bis (2-chloroaniline) (mboca) in new south wales, australia. J Occup Health. 59(3):296-303.
Shay E, De Gandiaga E, Madl AK. 2013. Considerations for the development of health-based surface dust cleanup criteria for beryllium. Crit Rev Toxicol. 43(3):220-243.
Smith JR, Birchall A, Etherington G, Ishigure N, Bailey MR. 2014. A revised model for the deposition and clearance of inhaled particles in human extra-thoracic airways. Radiat Prot Dosimetry. 158(2):135-147.
Soltani NS, Taylor MP, Wilson SP. 2021. Quantification and exposure assessment of microplastics in australian indoor house dust. Environ Pollut. 283:117064.
Stefaniak AB, Wade EE, Lawrence RB, Arnold ED, Virji MA. 2021. Particle transfer and adherence to human skin compared with cotton glove and pre-moistened polyvinyl alcohol exposure sampling substrates. J Environ Sci Health A Tox Hazard Subst Environ Eng. 56(5):585-598.
Sturm R. 2007. A computer model for the clearance of insoluble particles from the tracheobronchial tree of the human lung. Comput Biol Med. 37(5):680-690.

Sturm R, Hofmann W. 2006. A multi-compartment model for slow bronchial clearance of insoluble particles--extension of the icrp human respiratory tract models. Radiat Prot Dosimetry. 118(4):384-394.

Sturm R, Hofmann W. 2009. A theoretical approach to the deposition and clearance of fibers with variable size in the human respiratory tract. J Hazard Mater. 170(1):210-218.

Sugeng EJ, Leonards PEG, van de Bor M. 2017. Brominated and organophosphorus flame retardants in body wipes and house dust, and an estimation of house dust hand-loadings in dutch toddlers. Environ Res. 158:789-797.

Swartjes FA, Janssen PJ. 2016. Assessment of health risks due to arsenic from iron ore lumps in a beach setting. Sci Total Environ. 563-564:405-412.

Teunis PFM, Reese HE, Null C, Yakubu H, Moe CL. 2016. Quantifying contact with the environment: Behaviors of young children in accra, ghana. Am J Trop Med Hyg. 94(4):920-931.

Tsakirakis AN, Kasiotis KM, Anastasiadou P, Charistou AN, Gerritsen-Ebben R, Glass CR, Machera K. 2018. Determination of pesticide dermal transfer to operators and agricultural workers through contact with sprayed hard surfaces. Pest Manag Sci. 74(12):2858-2863.

Tsou MC, Hu CY, Hsi HC, Hu HJ, Ozkaynak H, Hseu ZY, Dang W, Bradham KD, Chien LC. 2018. Soil-to-skin adherence during different activities for children in taiwan. Environmental Research. 167:240-247.

Tulve NS, Egeghy PP, Fortmann RC, Xue J, Evans J, Whitaker DA, Croghan CW. 2011. Methodologies for estimating cumulative human exposures to current-use pyrethroid pesticides. J Expo Sci Environ Epidemiol. 21(3):317-327.

Van Dyke M, Martyny JW, Serrano KA. 2014. Methamphetamine residue dermal transfer efficiencies from household surfaces. J Occup Environ Hyg. 11(4):249-258.

Van Hooste W, Charlier AM, Rotsaert P, Bulterys S, Moens G, van Sprundel M, De Schryver A. 2010. Work-related helicobacter pylori infection among sewage workers in municipal wastewater treatment plants in belgium. Occupational and Environmental Medicine. 67(2):91-97.

Van Horne YO, Chief K, Charley PH, Begay MG, Lothrop N, Canales RA, Beamer PI. 2023. A community-based health risk assessment following the gold king mine spill: Results from the gold king mine spill dine exposure project. Exposure and Health.

Walker MD, Vincent JC, Benson L, Stone CA, Harris G, Ambler RE, Watts P, Slatter T, López-García M, King MF et al. 2022. Effect of relative humidity on transfer of aerosol-deposited artificial and human saliva from surfaces to artificial finger-pads. Viruses. 14(5).

Wang B, Xue M, Lv Y, Yang Y, Zhong J, Su Y, Wang R, Shen G, Wang X, Tao S. 2011. Cell absorption induced desorption of hydrophobic organic contaminants from digested soil residue. Chemosphere. 83(11):1461-1466.

Wang X, Zhao JC, Yang YC, Xu YK. 2022. Cross-regional dynamic transfer characteristics of liquid oil contamination induced by random contact in machining workshops in shanghai, china. Applied Sciences-Basel. 12(9).

Wang YL, Tsou MM, Pan KH, Özkaynak H, Dang W, Hsi HC, Chien LC. 2021. Estimation of soil and dust ingestion rates from the stochastic human exposure and dose simulation soil and dust model for children in taiwan. Environ Sci Technol. 55(17):11805-11813.

Watson AP, Armstrong AQ, White GH, Thran BH. 2018. Health-based ingestion exposure guidelines for vibrio cholerae: Technical basis for water reuse applications. Sci Total Environ. 613-614:379-387.

Weeks JJ, Hettiarachchi GM, Santos E, Tatarko J. 2021. Potential human inhalation exposure to soil contaminants in urban gardens on brownfields sites: A breath of fresh air? J Environ Qual. 50(3):782-790.

Weiss JM, Gustafsson A, Gerde P, Bergman A, Lindh CH, Krais AM. 2018. Daily intake of phthalates, mehp, and dinch by ingestion and inhalation. Chemosphere. 208:40-49.

Wensing M, Uhde E, Salthammer T. 2005. Plastics additives in the indoor environment--flame retardants and plasticizers. Sci Total Environ. 339(1-3):19-40.

Williams RL, Bernard CE, Dyk MB, Ross JH, Krieger RI. 2008. Measurement of transferable chemical residue from nylon carpet using the california roller and a new mega-california roller. Journal of Environmental Science and Health Part B-Pesticides Food Contaminants and Agricultural Wastes. 43(8):675-679.

Wu CC, Jiang YJ, Bao LJ, Zeng EY. 2022. Transfer of frictional contact derived phthalates from pad surface enhances dermal exposure. Environ Sci Technol. 56(18):12999-13007.

Wu Q, Oldi JF, Kannan K. 2011. Fate of perchlorate in a man-made reflecting pond following a fireworks display in albany, new york, USA. Environ Toxicol Chem. 30(11):2449-2455.

Xie MJ, Wu YX, Little JC, Marr LC. 2016. Phthalates and alternative plasticizers and potential for contact exposure from children's backpacks and toys. Journal of Exposure Science and Environmental Epidemiology. 26(1):119-124.

Xu F, Giovanoulis G, van Waes S, Padilla-Sanchez JA, Papadopoulou E, Magnér J, Haug LS, Neels H, Covaci A. 2016. Comprehensive study of human external exposure to organophosphate flame retardants via air, dust, and hand wipes: The importance of sampling and assessment strategy. Environ Sci Technol. 50(14):7752-7760.

Xue J, Zartarian V, Moya J, Freeman N, Beamer P, Black K, Tulve N, Shalat S. 2007. A meta-analysis of children's hand-to-mouth frequency data for estimating nondietary ingestion exposure. Risk Anal. 27(2):411-420.

Xue J, Zartarian VG, Ozkaynak H, Dang W, Glen G, Smith L, Stallings C. 2006. A probabilistic arsenic exposure assessment for children who contact chromated copper arsenate (cca)-treated playsets and decks, part 2: Sensitivity and uncertainty analyses. Risk Anal. 26(2):533-541.

Yang Y, Wang Y, Tan F, Zhang Z, Rodgers TFM, Chen J. 2020. Pet hair as a potential sentinel of human exposure: Investigating partitioning and exposures from opes and pahs in indoor dust, air, and pet hair from china. Sci Total Environ. 745:140934.

Yoshida-Ohuchi H, Shinohara N. 2020. Estimated internal exposure doses due to indoor radiocaesium contamination in residential houses after the fukushima nuclear accident. Sci Rep. 10(1):17212.

Yusuf I, Hansson E, Eriksson M, Roos P, Lindahl P, Pettersson HBL. 2023. Particle size dependent dissolution of uranium aerosols in simulated gastrointestinal fluids. Health Phys. 124(4):285-300.
Zartarian V, Xue J, Glen G, Smith L, Tulve N, Tornero-Velez R. 2012. Quantifying children's aggregate (dietary and residential) exposure and dose to permethrin: Application and evaluation of epa's probabilistic sheds-multimedia model. J Expo Sci Environ Epidemiol. 22(3):267-273.
Zartarian VG, Xue JP, Ozkaynak H, Dang W, Glen G, Smith L, Stallings C. 2006. A probabilistic arsenic exposure assessment for children who contact cca-treated playsets and decks, part i: Model methodology, variability results, and model evaluation. Risk Analysis. 26(2):515-531.
Zhang R, Li J, Wang Y, Jiang G. 2023. Distribution and exposure risk assessment of chlorinated paraffins and novel brominated flame retardants in toys. J Hazard Mater. 447:130789.
Zhang Y, Li X, Zhang H, Liu W, Liu Y, Guo C, Xu J, Wu F. 2022. Distribution, source apportionment and health risk assessment of phthalate esters in outdoor dust samples on tibetan plateau, china. Sci Total Environ. 834:155103.
Zhao C, Li JF, Li XH, Dong MQ, Li YY, Qin ZF. 2021. Measurement of polychlorinated biphenyls with hand wipes and matched serum collected from chinese e-waste dismantling workers: Exposure estimates and implications. Sci Total Environ. 799:149444.
Zhao E, Xiong X, Hu H, Li X, Wu C. 2023a. Phthalates in plastic stationery in china and their exposure risks to school-aged children. Chemosphere. 339:139763.
Zhao L, Cheng Z, Zhu H, Chen H, Yao Y, Baqar M, Yu H, Qiao B, Sun H. 2023b. Electronic-waste-associated pollution of per- and polyfluoroalkyl substances: Environmental occurrence and human exposure. J Hazard Mater. 451:131204.
Zheng K, Zeng Z, Huang J, Tian Q, Cao B, Huo X. 2022. Kindergarten indoor dust metal(loid) exposure associates with elevated risk of anemia in children. Sci Total Environ. 851(Pt 1):158227.
Zhong P, Zhang JQ, Xu DM, Tian Q, Hu TP, Gong XY, Zhan CL, Liu S, Xing XL, Qi SH. 2020. Contamination characteristics of heavy metals in particle size fractions from street dust from an industrial city, central china. Air Quality Atmosphere and Health. 13(7):871-883.
